# Gas Chromatography Mass Spectrometry (GC-MS) Quantification of Metabolites in Stool Using ^13^C Labelled Compounds

**DOI:** 10.3390/metabo8040075

**Published:** 2018-10-31

**Authors:** Oliver Gould, Ben de Lacy Costello, Amy Smart, Peter Jones, Angus Macmaster, Karen Ransley, Norman Ratcliffe

**Affiliations:** 1 Institute of Biosensor Technology, University of the West of England, Bristol BS16 1QY, UK; oliver.gould@uwe.ac.uk (O.G.); amy.smart@uwe.ac.uk (A.S.); 2 Indigo Science Ltd., Bristol BS7 9JS, UK; prhjones@gmail.com; 3 Givaudan UK Ltd., Ashford TN24 0LT, UK; angus.macmaster@givaudan.com (A.M.); karen.ransley@givaudan.com (K.R.)

**Keywords:** gas-chromatography mass-spectrometry, volatile compounds, quantification, carbon isotopes, stool

## Abstract

It has become increasingly important to qualitatively and quantitatively assess the volatile metabolites in a range of bodily fluids for use in monitoring health. There has been relatively little work on the quantitative analysis of compounds, particularly with respect to the effects of ethnicity or geographic location. A novel method for the quantification of compounds in stool using ^13^C labelled compounds as internal standards is presented. Using thermal desorption gas chromatography mass spectrometry, stool samples from 38 healthy volunteers were analysed. The ^13^C labelled compounds, acetone, ethyl butanoate, ethanoic acid, butanoic acid, 3-methylbutanoic acid, and indole, were added as internal standards. This process mimics the solubility characteristics of the compounds and thus the method was able to quantify the compounds within the solid stool. In total, 15 compounds were quantified: Dimethyl sulphide (26–25,626 ng/g), acetone (442–3006 ng/g), ethyl butanoate (39–2468 ng/g), ethyl 2-methylbutanoate (0.3–180 ng/g), dimethyl disulphide (35–1303 ng/g), 1-octen-3-one (12 ng/g), dimethyl trisulphide (10–410 ng/g), 1-octen-3-ol (0.4–58 ng/g), ethanoic acid (672–12,963 ng/g), butanoic acid (2493–11,553 ng/g), 3-methylbutanoic acid (64–8262 ng/g), pentanoic acid (88–21,886 ng/g), indole (290–5477 ng/g), and 3-methyl indole (37–3483 ng/g). Moreover, by altering the pH of the stool to pH 13 in conjunction with the addition of ^13^C trimethylamine, the method was successful in detecting and quantifying trimethylamine for the first time in stool samples (range 40–5312 ng/g). Statistical analysis revealed that samples from U.K. origin had five significantly different compounds (ethyl butanoate, 1-octen-3-ol, ethanoic acid, butanoic acid, pentanoic acid, and indole) from those of South American origin. However, there were no significant differences between vegetarian and omnivore samples. These findings are supported by pre-existing literature evidence. Moreover, we have tentatively identified 12 compounds previously not reported as having been found in stool.

## 1. Introduction

Numerous studies have been conducted which present volatile compounds (VCs) as potential biomarkers for gastrointestinal (GI) disease states [1,2,3,4]. For instance, Garner et al. showed VCs were significantly different in infected patients with *C. difficile*, and *C. jejuni* compared to healthy participants [2]. Similarly, a pilot study suggested potential VC biomarkers of cholera [1]. Other work has suggested the VCs emitted from stool can have potential in the diagnosis of irritable bowel syndrome (IBS) and inflammatory bowel disease (IBD) [3,4,5]. Currently, GI diseases are commonly diagnosed invasively, via blood tests, endoscopy, and biopsy, while VC analysis of stools is non-invasive and is potentially more economical. 

However, if VCs are to have real clinical utility, it is crucial that the healthy state volatilome is better understood. The gut is known to contain a vast and dynamic population of bacteria, with an estimated 100 trillion bacteria comprised of ca. 1000 species [6]. Currently, some 381 compounds have been identified from human stool, and this number may be unrepresentative considering over double this number of compounds have been identified in breath [7]. This discrepancy may simply be due to fewer studies attempting to identify compounds from stool rather than breath due to the increased complexity of sample gathering and difficulty in obtaining healthy stool samples. Determining the healthy gut volatilome is problematic as dietary alterations can both alter the composition of the microbiota and the metabolites produced [8]. For instance, Geypens et al. [9] investigated the effect of a high protein diet on VCs in stools, measuring the volatiles before and after a whey-protein supplemented diet in healthy volunteers. The study identified 120 VCs, 10 of which appeared or increased after the protein-rich diet, particularly dimethyl trisulphide and short-chain fatty acids (SCFAs). Silvester et al. showed that high protein meals can also contribute to an increase in the production of N-nitroso compounds and ammonia by intestinal flora [9]. 

Currently, the quantification of volatiles from biological samples are mostly restricted to real-time methods, such as selected ion flow tube mass spectrometry (SIFT-MS) [10]. However, the use of GC-MS in the quantification of volatile compounds has previously been reported using solid phase micro extraction (SPME) to sample latrine models [11]; 10 VOCs were quantified, with butanoic acid being found in the highest concentration with a range of 46.2–13,578 μg/g, whereas dimethyl sulphide was measured in the lowest concentration range of 0.02–2.1 μg/g [11].

Walton et al. quantitatively analysed VCs in the faecal headspace before and after treatment from patients with Crohn’s disease (CD), ulcerative colitis (UC), and irritable bowel syndrome (IBS), and healthy controls. Following treatment, only propanoic acid ethyl ester remained significantly different across the groups [12]. Similarly, Baranska et al. used breath samples to differentiate IBS patients from healthy controls using GC time-of-flight mass spectrometry. This study suggests a set of 16 biomarkers that could be used to not only predict IBS, but also monitor its progression [13]. 

Wang et al. analysed stool samples of autistic spectrum disorder (ASD) children and healthy, age-matched controls for short chain fatty acids, phenols, and ammonia. Those with ASD had higher concentrations of acids than the controls and higher faecal ammonia [14]. 

De Preter et al. used purge and trap GC-MS analysis to quantify VCs in dried stool samples using calibration curves with diethyl sulphide, 2,6-dimethylphenol, and 2-ethylbutyric acid used as internal standards. A total of 135 different VCs were reported with 22 compounds common to all volunteers. Butanoic acid was found in the highest concentration with a range of 5–500 mg/L and dimethyl sulphide was found in the lowest concentration with a range of 5–1000 μg/L [15].

GC-MS analysis of VOCs from the human body has typically provided qualitative data and limited work exists on the quantification of metabolites in human stool. However, a method has been developed based on using internal ^13^C labelled standards to calculate concentrations of key compounds in the solid stool sample and the headspace. This study used stool from participants of different nationalities and varying diets (vegetarian/omnivore) to assess the difference in VC composition. Knowing what constitutes a “healthy” profile across a range of geographic locations allows a better understanding of the deviations from this profile, which may be indicative of disease. No work has appeared to be published on VC analysis with pH alteration of stool. Altering the pH of stool to alkaline conditions was explored to allow quantification of some amine compounds.

This study qualitatively identified compounds associated with stool samples from healthy volunteers and thus adds knowledge to the established human volatilome.

## 2. Results

### 2.1. Qualitative Data from Unmodified Stools

Tables S1–S12 show the raw qualitative data from all 38 samples; in total, 174 distinct chromatographic peaks were recorded across these samples; the supplementary tables show these compounds separated into chemical class. The mean number of peaks above the noise threshold was 57 per sample with a range of 36–72. Of the total number of chromatographic peaks, 32 could not be identified by the NIST library and 30 were identified as unspecified terpenes. Table S13 shows retention indices (RI) values calculated from the experimental data and compared where possible to literature values. All RIs for the unidentified and terpene compounds have also been calculated. Table S13 also highlights which compounds have been validated using standards. 

### 2.2. Quantitative Data from Unaltered Stools

Table 1 shows the mean and standard deviation for each compound in nanograms per gram of stool for all samples. The raw data for each individual sample can be found in Table S14. Table 1 also shows the mean concentration (ng/g) and standard deviation for each compound separated by samples of UK and South American origin, as these were the two largest groups.

### 2.3. UK Samples versus South America Samples

Mann Whitney U tests revealed six compounds to be significantly different between UK and South American samples, and these are shown in Table 2.

Discriminant analysis using stepwise statistics (Wilks’ Lambda) calculated butanoic acid and ethyl butanoate as being the two significant grouping variables. 

**Figure 1:**
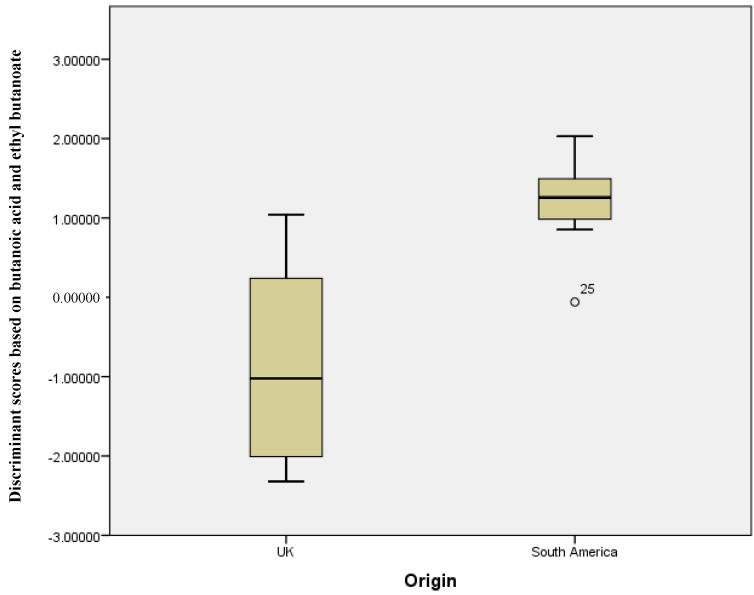
Discriminant scores’ box plots showing the differentiation between UK and South American stool samples (Wilks’ Lambda analysis showed butanoic acid and ethyl butanoate to be the significant grouping variables).

**Figure 2:**
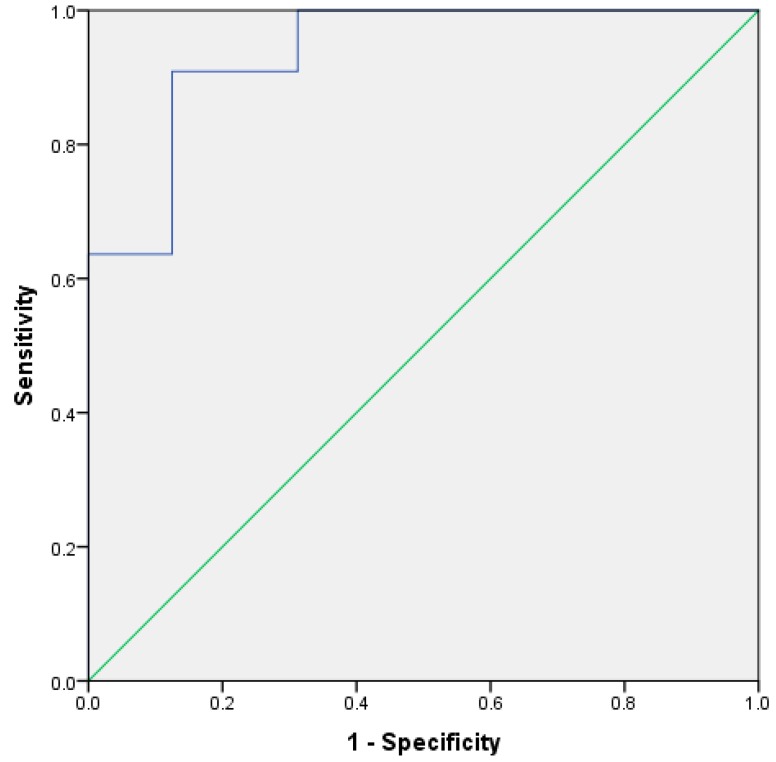
ROC curve based on leave-one-out cross validation discriminant scores, using butanoic acid and ethyl butanoate for different UK and South American stool samples.

Figure 1 shows a plot of the discriminant scores versus country of origin. There is a clear difference between the groups, with the South American sample set having a smaller range of scores versus the UK samples. This demonstrates that, with the exception of the outliers, the two means are very well separated. Figure 2 shows the resultant receiver operator curve (ROC) from the calculated discriminant scores; the area under the curve was 0.937 with a standard error of 0.044. This shows close to perfect separation of the U.K. and South American samples based on differences in the concentration of butanoic acid and ethyl butanoate. These two compounds alone can predict group membership.

### 2.4. Omnivore versus Vegetarian

**Figure 3:**
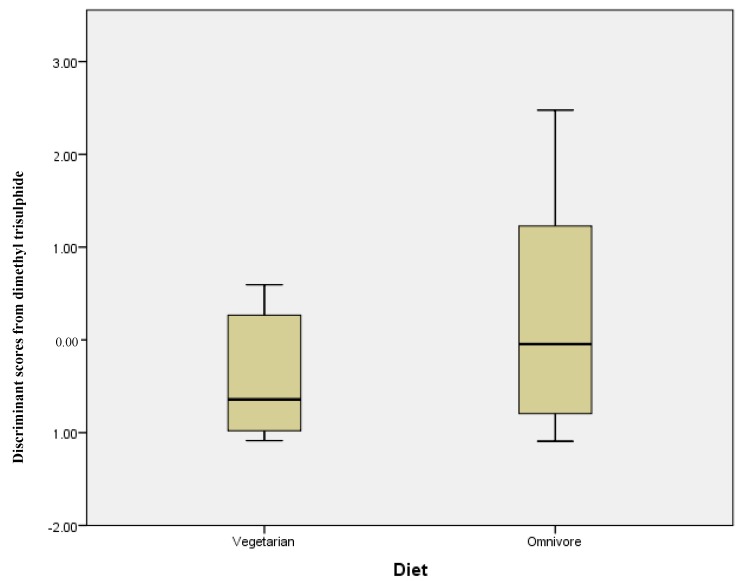
Discriminant scores’ box plots showing a lack of differentiation of stool samples from vegetarian and omnivore volunteers.

The same series of statistical tests were carried out comparing vegetarians and omnivores; Table 3 shows the mean concentration (ng/g) and standard deviation for each compound. However, in this instance, there was no statistically significant differences in those compounds analysed. Discriminant analysis using stepwise statistics (Wilks’ Lambda) calculated that the most significantly different compound was dimethyl trisulphide with a *p* = 0.055. The discriminant scores for this compound measured in the omnivore and vegetarian groups are plotted in Figure 3. While the range of discriminant scores is wider for omnivore participants, the two groups completely overlap, illustrating the lack of any significant difference between the two groups.

#### 2.4.1. Qualitative Data from Alkaline (pH 13) Stools

A total of 15 stool samples were analysed after the addition of sodium hydroxide to alter the pH to 13. A total of 133 chromatographic peaks were recorded across the 15 samples with a mean of 43 peaks per chromatogram; with the lowest number of peaks being 29 and the highest 53. Of the total number of peaks, 16 were unidentified and 43 were categorised as terpenes. Tables S15–S25 in the Supplementary Material shows the qualitative data separated by chemical class. 

#### 2.4.2. Quantitative Data from Alkaline (pH 13) Stools

Figure S1 shows the comparison between chromatograms of unaltered stool (Figure S1a) and the same sample with 5 mL of 0.1 M sodium hydroxide added (Figure S1b). As expected, the region containing the majority of the short chain fatty acids (retention time 7–18 min) has both less chromatographic peaks and smaller peak areas when the sodium hydroxide is added. However, Figures S1 and S2 clearly shows that when sodium hydroxide is added to make the stool alkali, the trimethylamine can be detected. In the unmodified stool (Figure S1a) there is no 58 *m*/*z* peak at the 1.94 min retention time, which is indicative of trimethylamine. The peak with a retention of 1.91 in Figure S1a does not have any clear matches in the NIST library. This peak is no longer visible when only the 58 *m*/*z* for trimethylamine is displayed as shown in the section of the chromatogram displayed in Figure S2a. Whereas the identical section of the chromatogram for the pH altered stool gives a peak at the earlier retention time with a library match for trimethylamine (Figure S2b).

## 3. Discussion

It is considered that some diseases could be linked to the microbiome [16]. There is a lack of knowledge of gut chemistry, due in part to the shear complexity of the stool mixture. Recently, hundreds of new compounds have been identified in the gut mainly due to the VC analyses in headspace studies, and much of this work is of a qualitative nature [4,17]. We have developed a method for quantifying key compounds from stool samples, which minimizes chemical alterations to the stool using ^13^C isotope labelled internal standards. It quantifies headspace concentrations and accounts for dissolved stool concentrations by comparing the distribution and relative recovery of isotope labelled compounds from the headspace above stool. 

We have also devised a method to quantify trimethylamine in stool by using isotope ^13^C labelled trimethylamine in conjunction with elevating the pH to 13. Although addition of base to stool samples is not common, Wang et al. also added sodium hydroxide to stool prior to the sample being centrifuged and filtered to measure lactic acid and SCFAs with high-performance liquid chromatography [18]. However, in the Wang et al. study, no healthy participants were used, and all the participants were premature infants. This study also differs from our study as only 1 mL of 10 mmol/L sodium hydroxide was added along with 5 mmol/L of crotonic acid, with no mention as to the effect these additions had on the pH. 

Walton et al. used TD-GC-MS, a similar method to that reported here, to analyse the headspace of stool samples. They found levels of acetone that were 142 ng/L whereas butanoic acid was 33 ng/L, 3-methylbutanoic acid was 7 ng/L, and Indole was 9 ng/L. Measuring the headspace concentration underestimates the level of free acid and other compounds, such as indole, in the stool, due to the relatively low concentrations portioning into the headspace. To determine the differences in quantification of the headspace concentrations versus the in-stool concentrations, headspace quantification was done on a subset of samples (16 UK samples). Although the levels of acetone, dimethyl sulphide, and other lower molecular weight, less water soluble, and lower boiling point compounds were equivalent to the values measured during the quantitation studies for the same samples (Table 1 and Table S14, UK participants), much lower amounts of acids and indole were determined if just pure headspace concentrations were measured (see Supplementary Table S26). Thus, the method we devised considered the headspace concentration vs. dissolved concentration, by dissolving a known ^13^C isotope with similar chemical properties into the stool and measuring headspace concentrations, but correcting for lower than expected recovery and applying this same correction factor to the levels detected (peak areas) of the non-isotope labelled naturally occurring compounds. 

As mentioned, fatty acids and indole concentrations in stool are disproportionately underestimated if just the headspace concentration is considered. The devised method does rely on distributing the ^13^C labelled compound throughout the stool sample. To ensure the best possible distribution, method development identified the use of a ball bearing to homogenise the thawing stool sample plus isotope labelled standard. We measured recoveries of isotope labelled compounds and non-labelled standards from an empty vial. This showed 100% recovery for acetone, but as expected, less than 100% for indole and the fatty acids. The recovery for labelled standards and non-labelled standards was almost identical as expected due to the similar chemical properties. The relative recovery of compounds is not used in the final calculation as lower amounts of certain isotope labelled compounds (acids and indole) are expected to be recovered—but this is balanced by the correction that would have to be applied to account for the lower amounts of naturally occurring compounds from the stool that would be measured (recovered) vs. the actual concentration in stool. 

Extensive method development was undertaken using standard mixtures containing 18 compounds previously identified in stool to optimise the chromatography method and automated thermal desorption (ATD) tube loading. Prior to this, a number of different solvents had been used for the standard mixture and methanol was selected due to its compatibility with a wide range of compounds, ease of removal from ATD tubes when loading calibration standards, and minimal solvent peak, which did not interfere with the compounds of interest in unmodified stool.

The method relies on correcting recovered values based on the ^13^C standard recovery. This gives a more realistic quantitation for the amount of certain compounds in stool. However, if the naturally occurring compounds of interest are below the limit of detection for the headspace analysis method, then there is no way to apply the correction to ascertain the actual concentration of the compound in stool. Therefore, a solvent extraction method, which does not rely on working close to the limit of detection, may have enabled quantitation of the full range of samples. Prior to developing the headspace-based method reported here, a solvent extraction method had been developed for quantifying selected compounds in stool. However, it proved difficult to obtain a reliable solvent extraction method that enabled simultaneous quantitation of the compounds of interest, which span a broad range of chemical classes. 

Wang et al. utilized a vacuum distillation process to isolate the short chain fatty acids (SCFAs) in stool [14]. These studies identified higher levels of SCFA than the method reported here, with levels in the high μg/g range compared to this study where values were in the low μg/g range. Wang et al. obtained median values of 3705 μg/g for ethanoic acid, 1756 μg/g for butanoic acid, and 285 μg/g for 3-methylbutanoic acid. These values are higher than those reported here due to the methodology used, whereby both the free acid and anionic forms would be extracted and analysed. 

De Preter et al. freeze dried their stool samples before they were salted with sodium sulphate and acidified with sulphuric acid [15]. Acidification of stool samples is a reasonably common technique as detailed in a comprehensive review of SCFA analysis via GC and other methods [19]. Their reported values for the SCFAS measured (ethanoic acid, butanoic acid, and 3-methylbutanoic acid) were in the high μg/g range in agreement with the studies of Wang et al. detailed above [14]. Interestingly, de Preter also quantified dimethyl sulphide and found levels of 0–402 ng/g whereas this study identified DMS at a mean concentration of 3058 ng/g with a range of 26–25,626 ng/g. So, there is fairly good agreement between the two different analysis methods for quantifying volatile sulphur compounds, and this is because the alteration of the chemistry does not affect the quantification of sulphides in the same way as SCFAs. De Preter also quantified indole and found levels ranging from 24 μg/g to 44 μg/g whereas this study found mean values of 3 μg/g. Therefore, there is fairly good agreement that can be explained by the different effects altering the stool chemistry has on quantifying indole vs. quantifying SCFAs.

As mentioned, by the action of acidifying stool, both the free acid and the previously anionic form is measured. However, in the gut, the acid exists as free acid, but also more so in the anionic form [20] (with the counter cation being H^+^, metal cations, and, to a lesser extent, ammonium ions). Our contention is that quantification of the free SCFA concentration in addition to the total SCFA concentration is important to know in a study of the gut chemistry. The amount of free acid will dictate the amount of acid detectable in the headspace and thus the amount available for potential diagnostic purposes [21]. 

Preliminary work on the basification of stool was reported. The unmodified stools yielded a mean of 174 peaks across 38 samples with a mean of 57 per sample (range 36–73). The addition of the sodium hydroxide reduced both values significantly to 133 and 43 (range 29–53), respectively. Moreover, as demonstrated in Figure S1a,b, after the first 10–12 min, the frequency and size of the chromatographic peaks notably reduces. By making the stool alkali, we have shown that it is possible to not only detect, but also quantify, trimethylamine, which is likely to be a result of the conversion of protonated trimethylamine to trimethylamine in the high pH conditions. Figure S2b shows a clear peak with an RT of 1.94 after the sodium hydroxide is added, which is identified by the NIST library with a match and reverse-match at 999 and 992, respectively. Figure S2a shows the same sample with unmodified stool in which the 1.94-min peak is barely visible. Lin et al. found that in their latrine models, the amine smell became more prominent as the pH increased to 9 [11]. They also found that trimethylamine could not be detected from their field samples, and this could be due to the presence of acids in the sample protonating the amine compounds [9], resulting in a lack of free trimethylamine within the samples. Simenhoff et al. suggested that secondary and tertiary amines were in high levels on the breath of patients with end stage renal disease [22]; a finding that was also supported by Davis, Spanel, and Smith [23]. Moreover, ammonia has been associated with hepatic encephalopathy [24]. Thus, improving amine detection techniques could have clinical utility in the future. While we were unable to quantify any other amine compounds in this instance, we are confident that with further method development we should be able to quantify more amine compounds. We were able to identify methylamine in one of the stools modified to pH13, and this peak eluted as a shoulder to the methanol solvent peak; this is suggestive that methylamine may be in other samples, but co-elutes with the solvent peak. Thus, developing the method further to reduce or eliminate this solvent peak, for instance, by increasing the tube collection purge time to drive off more methanol, may reveal more amine compounds. Table 4 shows the differences in the number of compounds detected for each chemical class between the unaltered and alkaline stool samples. There were fewer compounds detected across all the chemical classes, with the exception of nitrogen containing compounds, in the alkaline stool; not surprisingly, the largest percentage decrease in the number of compounds came from the acids with a 70% decrease. The least change came from the esters, which only decreased by 17% following pH alteration. As expected the only class of compounds that showed an increase in numbers were the nitrogen containing compounds. 1,6-Octadien-3-ol-3,7-dimethyl-2-aminobenzoate was detected in the unmodified stool, but was not detected in the alkaline stool. Trimethylamine, dimethylamine, and acetonitrile (dimethylamino) were detected in the alkaline stool, but not in the unmodified stool. Acetonitrile, indole, and 3-methyl indole were seen in both unmodified and pH modified stool. 

The origins of a majority of the esters could arise from reactions between the alcohols and acids reported here. A homologous series of alcohols from ethanol to octanol was found, and from ethanoic acid to heptanoic acid, in agreement with previous work [25], and their reaction would produce many straight chain esters. Branched chain esters can similarly be explained by a reaction of, for example, 3-methylbutanoic acid with alcohols. Interestingly, a large number of methyl esters (nine in total) were found although no free methanol was observed. It may be that that the body, or bacterial enzymes, particularly “trap” methanol as esters, reduce the methanol’s toxic effects on cells.

The statistical tests on the gathered data showed that five compounds were in significantly different quantities in stool gathered from UK and South American participants; significantly, these participants were temporarily living in the UK with little exposure to a UK diet due to the short duration of their time in the UK. Moreover, using one cross validation, we were able build an example model using butanoic acid and ethyl butanoate to differentiate UK and South American samples; albeit, this was based on a small dataset. However, there was very little overlap between the two groups. In many clinical studies, there is little attention paid to the ‘healthy’ participants; however, developing a comprehensive understanding of how healthy samples can differ from population to population could be significant in the development of VC based diagnostics. For example, a stool volatile based diagnostic test that has high accuracy in the UK may not exhibit the same accuracy in a South American population. The results presented here do seem to suggest that further work is required to assess the difference in volatile profiles of healthy participants of different geographical origins.

A 2010 study compared the microbiota of children from Burkina Faso with children from the European Union (EU) [26]. This team suggested that there was a significant difference in the composition of gut microbiota between the two groups; they proposed the increased sugar, animal fats, and general calorie dense foods as the reason for this difference [26]. A difference in microbiota composition will inevitably lead to differences in the faecal volatilome. Similarly, a research team from China assessed the gut microbiota of 314 healthy participants of different geographical origins within China. This group was also able to determine differences in the composition of gut microbiota from these geographical origin groups; interestingly, this study was unable to determine any difference as a result of lifestyle. However, importantly, the team state they did not process the necessary dietary information to make any inferences on the role diet plays in the formation of microbiota [27]. A 2006 study examined the gut microbiota of four different European countries; in this instance, very few differences were noted as a result of country of origin, however, this study suggested that other factors, such as age and gender, conferred significant differences [28]. Taken together, there is sufficient evidence to say that there are a number of variables that can influence the gut microbiota and, in turn, the associated volatilome. 

Comparing the quantities of the compounds in omnivore samples versus vegetarian samples revealed no significant differences between the two groups, as evidenced by the discriminant scores plot shown in Figure 3. There have been numerous publications that suggest why this might be the case, for instance, Kabeerdoss et al. compared the microbiota of female southern Indian omnivores and vegetarians. This groups found that, with the exception of *Clostridium* cluster XIVa and some butanoate producing bacteria, which were more prevalent in omnivores, the groups were very similar [29]. While our results showed no significant difference due to the large standard deviations, the butanoic acid, ethyl butanoate, and ethyl 2-methylbutanoate were all higher in the omnivore group versus the vegetarian group. Van Faassen et al. demonstrated that while stool mass and frequency was higher in vegetarians versus omnivores, the pH of the stool was not significantly different; this was attributed to both vegetarians and omnivores having similar calcium intake [30]. In a recent 2015 study, Ferrocino et al. assessed the gut microbiota of 153 healthy participants from five different regions of Italy. This group also found that significant differences in gut microbiota could be attributed to region of origin rather than dietary habits (vegetarian/omnivore) [31].

A 2014 review of the healthy human volatilome found 381 distinct compounds from human stool [25]. Overall, we were able to identify 106 distinct compounds; moreover, we have tentatively identified additional compounds that have not previously been reported in the literature from stool samples of healthy participants (see Table 5). However, 3-Methyl-2-butanone has previously been reported in the urine samples of healthy individuals [31]. The compound, 2,4-dithiapentane, has been associated with white truffles [32] and truffle oil [33], and is likely to be directly derived from diet. Cyclohexanol, 5-methyl-2-(1-methylethyl)-(1a,2b,5a) is a none verified isomer of menthol [34], and 4-isopropyl benzaldehyde is better known as a cuminaldehyde and both of these compounds are found in food [35]. Table 6 shows a further three compounds that were identified in the alkali treated stool samples that have not previously been reported in the current literature concerning volatile compounds emanating from stool of healthy individuals, but acetonitrile (dimethylamino) was identified in the headspace above stool of patients with *C. difficile* [2]. It is important to understand more about the volatilome if volatile compounds are going to prove useful in monitoring health. It may be particularly important to understand the gut derived volatilome both directly and indirectly due to the important role the microbiome plays in human health. Table 5 and Table 6 show the retention indices for each compound calculated from the sample versus the literature values, in all cases where literature values exist or samples matched closely. Table S14 shows the RI values for all chromatographic peaks detected across all samples and compares those to the literature. In the vast majority of cases, our experimental value is in-line with those in the literature. Figures S3–S14 in the Supplementary Materials shows the mass spectra for each of the compounds in Table 5 and Table 6. Trimethylamine was checked against the standards used for quantification. Future work will include verifying all of these compounds with chemical standards.

## 4. Materials and Methods

### 4.1. Instrumentation and Separation Methodology

A Clarus 600 gas chromatograph (GC) and Clarus 600 T mass spectrometer (MS) (Perkin Elmer, Buckinghamshire, UK) was used for all the experiments described. 

The GC used a 30 m × 0.25 mm SOLGEL-WAX 0.25 μm column (Trajan scientific Europe Ltd.). The GC method started at 40 °C with a 4-min hold, then ramped at a rate of 8 °C per minute to 240 °C, with a final 4-min hold, and a total run time of 33 min. The MS was set to scan *m*/*z* 29–450 with electron ionisation selected.

The automated thermal desorption (ATD) unit was a Turbo matrix 350 (Perkin Elmer, Buckinghamshire, UK). TD tubes were filled with Tenax TA 26 mg and Sulficarb 68 mg absorbents (Markes International Ltd., Llantrisant, UK). The valve was set to 215 °C with a tube temperature of 315 °C. The transfer temperature was set to 300 °C with a trap rate of 99 °C/second; the trap low and high was set to −20 °C and 320 °C, respectively. The dry purge time was 5 min and the desorb time was 10 min with a 1.2 mL/min column flow rate. The outlet split was 2 mL/min and the desorb was 180 mL/min; the inlet split was 25 mL/min with a 50 mL/min dry purge. The heated purge temperature was 50 °C.

### 4.2. Loading the Thermal Desorption (TD) Tubes

Thermal desorption (TD) tubes were loaded using an adapted single shot heated auto sampler from an SRI GC instrument. This has a temperature controlled heating block. In the modified version, 2 needles pierce the headspace vial, one that allows the nitrogen purge gas (BOC UN1066 99.998%) to enter the vial, and the other, which is securely attached to the TD tube with brass fittings (Swagelok). The vials were 10 mL glass headspace vials with a screw top phenolic cap and PTFE/silicone septa (Supelco, Bellefonte, PA, USA). The SRI GC instrument uses an EPC valve to control the purge gas flow, which was set to 80 mL/min and flowed through the headspace vial containing the sample. The purge gas is flowed through the vial and out through the TD tube for 2 min; the flow rate through the tube was checked each time using a Perkin Elmer PE 1000 flow meter (Perkin Elmer, Buckinghamshire, UK). 

1 μL standards dissolved in methanol were injected onto the TD tube using a 1 μL syringe (SGE analytical science, Ringwood, Australia) (see standard solutions section for details on solutions used) and loading rig (Markes International Ltd., Llantrisant, UK). Following the injection, nitrogen gas (BOC UN1066 99.998%) was flowed through the tube at 80 mL/min for 2 min to remove excess solvent prior to analysis. Flow was measured each time using a Perkin Elmer PE 1000 flow meter (Perkin Elmer, Buckinghamshire, UK). 

Once the tube was loaded, it was added immediately to the ATD unit carousel and analysed within 30 min using the ATD-GC-MS.

### 4.3. Standard Solutions

Two solutions were used for this work. Table S27 shows solution 1, and ^13^C labelled compounds, which were used as internal standards in the samples and for the calibration curves (see Section 2.4 calibration curves). Table S28 shows solution 2, made of non-labelled compounds used for calibration curves. Both solutions were made up in methanol HPLC grade (Sigma Aldrich Company, Gillingham, UK) and stored at 4 °C. 

### 4.4. Calibration Curves

5 mL of solution 1 was sequentially diluted by factors of 10 and 100, using methanol. 0.5 μL of the solutions 1 and 2, and the sequentially diluted solutions, were injected on to TD tubes following the steps described in Section 4.2 of loading TD tubes. These were then analysed on the GC-MS, and the peak areas were recorded and noted for each compound. Calibration curves were created for each compound; none of the compounds in solution 1 or solution 2 yielded an r^2^ of less than 0.99. Retention indices for all the standards were compared to that of the literature, and in all cases, the values obtained from our data matched the literature values.

### 4.5. Sample Preparation

Stool samples were collected from 38 healthy volunteers, age range of 18–60 years, with full ethical consent (research ethics committee reference 14/NE/0029); these were immediately refrigerated (4 °C) on arrival and processed in a microbiological safety cabinet within 4 h. Samples were initially collected in aluminium containers, L 20 cm × W 11.5 cm × H 4 cm. Processing involved taking 6–8 aliquots weighing 3 g from each sample and placing them into 10mL headspace vials (Supelco), which were then frozen at −20 °C. The ethnic origin and the omnivore/ vegetarian status of the samples is shown in Table 7.

For analysis, the sample vial was removed from the freezer and immediately the screw cap was removed and a steel ball bearing (0.5 cm diameter, weighing approximately 0.5 g) was added and the vial recapped. Next, 1 μL of solution 1 (see standard solutions section) was injected through the septa using a 1 μL syringe (SGE analytical science). The vial was then inserted into the TD loading rig heating block at 30 °C for 10 min, manually shaken for ca*.* 1 min, and returned to the block. After a further 10 min, the vial was shaken for a second time; then, it was returned to the block for a further 10 min, giving a total 30 min of heating time (see Section 4.2 loading TD tubes section for details).

### 4.6. Trimethylamine Quantification in Ph13 Stool Samples

Trimethylamine solution standards were made separately. 25 mg of ^13^C trimethylamine (99% Cambridge Isotope Laboratories Ltd., Tewksbury, MA, USA) was dissolved into 25 mL of methanol to give a final concentration of 1 mg/mL (most concentrated), and this 1 mg/mL solution was sequentially diluted to 0.1 mg/mL and 0.01 mg/mL. Trimethylamine, 400 mg (25 wt % in water, Sigma Aldrich Company, Gillingham, UK) was dissolved in 100 mL methanol to give the same 1 mg/mL concentration, which was again used as the most concentrated standard. This was diluted 10 fold to give 0.1 mg/mL and 0.01 mg/mL. These solutions were used to create calibration curves of the mass of compounds (*x*-axis) versus peak area recovered from the chromatogram (*y*-axis) (see calibration curves Section 4.4).

The same sample processing took place as described in the sample preparation section above; however, in the case when the ball bearing was added to the sample on removal from the freezer, 5 mL of aqueous 0.1 M sodium hydroxide (Fisher Scientific, Loughborough, UK) was also pipetted into the sample. The pH of the stool samples were checked post analysis with indicator paper. A total of 15 stool samples were run using this method.

### 4.7. Analysis

#### 4.7.1. Qualitative Analysis

A signal threshold of 3 times the noise was set for all chromatograms. All peaks were searched manually using the NIST library (NIST 08). Compounds with a match and reverse match above 800 were identified. If the match and/or reverse match was under 800, then the peak was listed as unidentified. Terpene and siloxane compounds were difficult to assign structures to and thus were listed under the chemical class name. 

#### 4.7.2. Mass Calculations Using ^13^C Labelled Compounds

Using solution 1, the following compounds were quantified: Dimethyl sulphide, acetone, butanoic acid ethyl ester, 2-methylbutanoic acid ethyl ester, dimethyl disulphide, 1-octen-3-one, dimethyl trisulphide, 1-octen-3-ol, ethanoic acid, butanoic acid, 3-methylbutanoic acid, pentanoic acid, indole, and 3-methylindole. Peak areas of the quantified compounds were corrected using the recoveries of the ^13^C labelled compounds (the calculations used for this are detailed in the Supplementary Materials calculations for the quantification of compounds section). 

#### 4.7.3. Statistical Analysis

Basic descriptive statistics (mean and range numbers of chromatic peaks) were carried out on the qualitative data. The differences between samples (e.g., UK origin versus South American origin) have been discussed. Also discussed is the difference in chromatic peaks both qualitatively and quantitatively for unmodified stool versus pH 13 stool. 

The 2 largest groups of participants with different geographical origins were from the UK and South America, thus these groups were selected for comparison. Omnivore and vegetarian groups from the entire cohort of participants were also compared. Descriptive statistics, such as the mean, standard deviation, and the ranges, were carried out in the first instance. To compare sample groups, a Mann Whitley U test was carried out before a discriminant analysis using stepwise statistics (Wilks’ Lambda). Leave-one-out cross validation was used to calculate an ROC curve. All statistical analysis was carried out using IBM SPSS statistics version 24.

## 5. Conclusions

We have presented a method for the analysis of stool samples whereby the addition of ^13^C compounds has allowed us to quantify a range of VOCs in stool: Dimethyl sulphide (26–25,626 ng/g), acetone (442–3006 ng/g), ethyl butanoate (39–2468 ng/g), ethyl 2-methylbutanoate (0.3–180 ng/g), dimethyl disulphide (35–1303 ng/g), 1-octen-3-one (12 ng/g), dimethyl trisulphide (10–410 ng/g), 1-octen-3-ol (0.4–58 ng/g), ethanoic acid (672–12,963 ng/g), butanoic acid (2493–11,553 ng/g), 3-methylbutanoic acid (64–8262 ng/g), pentanoic acid (88–21,886 ng/g), indole (290–5477 ng/g), and 3-methyl indole (37–3483 ng/g). Moreover, by altering the pH of the stool to pH 13, in conjunction with the addition of ^13^C trimethylamine, we have also been able to detect and quantify trimethylamine for the first time (range 40–5312 ng/g). We were able to gather stool samples from participants of different countries of origin, which allowed us to compare the quantities of compounds from samples of UK origin with those of South American origin. Using a Mann Whitney U test, five compounds, ethyl butanoate, 1-octen-3-ol, ethanoic acid, butanoic acid, pentanoic acid, and indole, were calculated to be significantly different between South American and UK samples. Wilks’ Lambda analysis showed that butanoic acid and ethyl butanoate could be used to differentiate the two groups. This has important implications for future studies looking to develop diagnostic tests based on VCs, especially where these diagnostics are not based on distinct markers of disease, but on changes in a number of VCs that are commonly observed in healthy individuals. However, no significant differences between omnivores and vegetarians were observed in this study, in agreement with previous studies. Additionally, we have been able to tentatively identify 15 compounds that have not previously been reported from stool samples. This data adds to the understanding of the human volatilome.

## Figures and Tables

**Table 1:** The mean concentration (ng/g) and standard deviation for compounds quantified from the stool of healthy participants using GC-MS. The mean concentration (ng/g) and S.D. of each compound from the UK and South American participants has also been compared.

	Origin	Number of Samples	Number of Times Detected	Mean (ng/g)	Std. Deviation (ng/g)	Range (ng/g)
Dimethyl sulphide CAS 75-18-3	Total	38	37	3058	4531	25–25,626
	UK	16	16	2474	2913	26–8637
	South America	11	10	2890	2780	621–8079
Acetone CAS 67-64-1	Total	38	38	1481	626	442–3005
	UK	16	16	1335	614	442–2521
	South America	11	11	1652	487	819–2377
Ethyl butanoate CAS 105-54-4	Total	38	21	352	659	39–2468
	UK	16	14	424	714	39–2468
	South America	11	3	121	263	118–828
Ethyl 2-methylbutanoate CAS 7452-79-1	Total	38	12	8	29	0.34–180
	UK	16	10	16	44	0.3–180
	South America	11	2	4	12.8	9–42
Dimethyl disulphide CAS 624-92-0	Total	38	38	479	406	35–1302
	UK	16	16	301	275	48–864
	South America	11	11	583	445	36–1313
1-Octen-3-one CAS 4312-99-6	Total	38	1	0.31	2	12
	UK	16	1	0.74	3	12
	South America	11	0	0.00	0	Not detected
Dimethyl trisulphide CAS 3658-80-8	Total	38	38	132	116	10–409
	UK	16	16	81	69	10–253
	South America	11	11	156	119	13–364
1-Octen-3-ol CAS 3391-86-4	Total	38	13	4	13	0.4–58
	UK	16	10	11	18	1–58
	South America	11	3	0.35	0.72	0.4–2
Ethanoic acid CAS 64-19-7	Total	38	38	8756	2246	672–12,963
	UK	16	16	7400	2607	672–11,343
	South America	11	11	9583	1411	7968–12,963
Butanoic acid CAS 107-92-6	Total	38	38	7556	2310	2493–11,553
	UK	16	16	5783	2160	2493–8376
	South America	11	11	8740	800	7043–9953
3-Methylbutanoic acid CAS 503-74-2	Total	38	36	2726	2210	63–8262
	UK	16	16	1363	1029	64–3602
	South America	11	10	3391	2555	220–6885
Pentanoic acid CAS 109-52-4	Total	38	37	4443	4374	88–21,886
	UK	16	16	2078	1720	88–5970
	South America	11	11	7494	5879	946–21,886
Indole CAS 120-72-9	Total	38	38	3717	1119	290–5477
	UK	16	16	3002	797	1508–4309
	South America	11	11	4174	649	3286–5477
3-Methyl-1*H*-indole CAS 83-34-1	Total	38	38	1323	776	37–3483
	UK	16	16	1340	799	425–3054
	South America	11	11	1703	777	636–3483
Trimethylamine CAS 75-50-3	Total	15	15	241	157	40–920.5

**Table 2:** Statistical analyses showing significant differences (95% significance level) for six compounds using Mann Whitney U tests to determine differences in the quantities of compounds measured in stool from UK and South American participants.

Compound	Sig.
Ethyl butanoate	0.023
1-Octen-3-ol	0.034
Ethanoic acid	0.011
Butanoic acid	0.000
Pentanoic acid	0.001
Indole	0.000

**Table 3:** The mean concentration (ng/g) and measured concentration range (ng/g) for each compound in the omnivore and vegetarian diet groups.

	Diet	Number of Samples	Number of Times Detected	Mean (ng/g)	Std. Deviation (ng/g)	Range (ng/g)
Dimethyl sulphide 75-18-3	Vegetarian	14	14	4182	6818	91–25,626
	Omnivore	24	23	2403	2354	26–8089
Acetone CAS 67-64-1	Vegetarian	14	14	1285	522	442–2287
	Omnivore	24	24	1595	663	453–3006
Ethyl butanoate CAS 105-54-4	Vegetarian	14	7	243	511	39–1659
	Omnivore	24	14	415	735	40–2468
Ethyl 2-methylbutanoate CAS 7452-79-1	Vegetarian	14	4	1	4	0.3–13
	Omnivore	24	8	12	37	8–180
Dimethyl disulphide CAS 624-92-0	Vegetarian	14	14	355	335	37–1058
	Omnivore	24	24	553	432	35–1303
1-Octen-3-one CAS 4312-99-6	Vegetarian	14	1	0.85	0	12
	Omnivore	24	0	0.00	0	Not detected
Dimethyl trisulphide CAS 3658-80-8	Vegetarian	14	14	85	69	11–199
	Omnivore	24	24	160	130	10–410
1-Octen-3-ol CAS 3391-86-4	Vegetarian	14	6	8	14	2–38
	Omnivore	24	7	3	12	0.4–58
Ethanoic acid CAS 64-19-7	Vegetarian	14	14	8799	2155	4298–12,233
	Omnivore	24	24	8731	2343	672–12,963
Butanoic acid CAS 107-92-6	Vegetarian	14	14	7452	2938	2493–11,553
	Omnivore	24	24	7618	1922	2681–9633
3-Methylbutanoic acid CAS 503-74-2	Vegetarian	14	14	2319	1974	64–5821
	Omnivore	24	22	2965	2344	220–8262
Pentanoic acid CAS 109-52-4	Vegetarian	14	14	3123	2841	88–10,216
	Omnivore	24	23	5213	4953	730–21,886
Indole CAS 120-72-9	Vegetarian	14	14	3340	1505	290–5477
	Omnivore	24	24	3938	772	2342–5070
3-Methyl-1*H*-indole CAS 83-34-1	Vegetarian	14	14	1160	633	37–2267
	Omnivore	24	24	1419	846	404–3483

**Table 4:** Comparison of compounds found across different classes of chemicals in unmodified stool and the stool modified to pH 13.

Chemical Family	Unmodified	pH Modified	Percentage Decrease (Unmodified to pH13 Modified)
Aldehydes	6	3	50%
Esters and thioesters	36	30	17%
Ketones	12	7	42%
Alcohols	20	11	45%
Acids	10	3	70%
Nitrogen containing	4	6	50%
Sulphides	8	3	63%
Aromatic compounds	6	5	29%
Miscellaneous	2	0	100%
Terpenes	30	22	40%
Unidentified	32	16	50%

**Table 5:** Details of 12 compounds identified from the headspace above stool samples from healthy individuals that have not been previously reported in the literature [31].

CAS	Compound	Retention Time (min)	Retention Indices Sample	Retention Indices Literature
563-80-4	3-Methyl-2-butanone	4.56	972	970 [36]
556-24-1	3-Methylbutanoic acid methyl ester	5.49	1017	1024 [37]
97-87-0	Propanoic acid 2-methylbutyl ester	8.18	1152	1154 [37]
539-90-2	Butanoic acid 2-methylpropyl ester	8.45	1158	11,152 [37]
141-06-0	Pentanoic acid propyl ester	9.73	1215	1200–1233 [38]
1618-26-4	2,4-Dithiapentane	10.6	1260	1260 [39]
2313-61-3	2-heptanol	11.66	1308	1315.3 [40]
15356-70-4	5methyl-2-(1-methylethyl)-(1a,2b,5a) (Menthol)	16.9	1631	1630.4 [40]
55012-32-3	4-Isopropyl benzaldehyde (cuminaldehyde)	18.91	1783	1781.4 [40]

Bianchi et al. evaluated the retention indices for 250 compounds based on a polar column similar to that used to carry out our work [38]. Babushok, Linstrom, and Zenkevich reported the retention indices for 505 compounds frequently occurring in plant essential oils [40].

**Table 6:** Three compounds, previously unreported in the literature [31], identified from the headspace of alkaline treated stool samples.

CAS	Compound	Retention Time (min)	Retention Indices Sample	Retention Indices Literature
75-50-3	Trimethylamine	1.95	-	-
108-64-5	3-Methyl butanoic acid ethyl ester	6.48	1074	1068 [38]
926-64-7	Acetonitrile (dimethylamino)	10.02	1230	1243 [41]

**Table 7:** A breakdown of the participants by country of origin and omnivore/vegetarian status.

Number of Participants	Ethnic Origin	Omnivore	Vegetarian
16	UK	8	8
11	South America (Brazil, Mexico)	10	1
3	Mainland Europe (Czech Republic, Latvia, and Spain)	1	2
5	Asia (China, Vietnam, Iran)	4	1
3	Africa (Maldives, Nigeria)	1	2

## Supplementary Materials

The following are available online at http://www.mdpi.com/2218-1989/8/4/75/s1. Tables S1–S12 containing all found chromatographic peaks sorted by chemical family and retention time, also the peak area for each peak is shown. Table S13 shows the experimental retention indices for all the compounds and compares them to the literature values. Table S14 showing the calculated masses of 13 compounds found in stool. Tables S15 to S25 show all of the chromatographic peaks found in the stool with added sodium hydroxide, sorted by chemical family and retention time. Table S26 shows the quantification of compounds from the headspace. Tables S27 and S28 show the chemicals and concentrations along with supplier of the compounds used in the calibration standards. Figures S1 and S2 detail the difference in the unaltered versus the pH 13 stool and demonstrate the appearance of trimethylamine. Figures S3 to S14 show the mass spectra for the compounds not previously identified in stool samples. Also detailed are the calculations for the quantification of compounds.
