# Supplementary material for: Gas Chromatography Mass Spectrometry (GC-MS) Quantification of Metabolites in Stool Using 13C Labelled Compounds
Source: Metabolites. 2018 Oct 31;8(4):75. doi: 10.3390/metabo8040075 (PMC6316270; doi:10.3390/metabo8040075)
Supplement: Supplementary file 1 [file metabolites-08-00075-s001.pdf]

Supplementary materials

S1a)

CAS	Aldehydes	Retention time (mins)	1	2	3	4	5	6	7	8	9	10	11	12
75-07-0	Acetaldehyde	2.16	169.44		230.78	72	53.08	166.03	97.55		91.36	81.08	260.13	82.12
123-38-6	Propionaldehyde	2.43												
123-72-8	Butyraldehyde	3.10		12.26	4.52	24.8	10.74	9.49		4.47			10.25	
66-25-1	Hexanaldehyde	6.76	9.23	10.2	8.89	19.19	18.18	37.13	14.9	12.28		9.52	15.09	17.01
100-52-7	Benzaldehyde	15.05	2.72	6.52	2.34	4.97	3.33	1.95	1.6		2.29	2.06	2.25	
55012-32-3	Isopropyl benzaldehyde	18.91										4.24		

S1b)

CAS	Aldehydes	Retention time (mins)	13	14	15	16	17	18	19	20	21	22	23	24
75-07-0	Acetaldehyde	2.16	173.9	65.69	143.82	31.3	156.33	170.17	60.17	30.89	39.56	42.08	65.72	30.64
123-38-6	Propionaldehyde	2.43							5.29					
123-72-8	Butyraldehyde	3.10			5.71			10.18	9.12	7.26			10.1	3.56
66-25-1	Hexanaldehyde	6.76	12.85	105.43	8.35	9.07	63.28	39.54	4.58	8.77	6.63	8.6	2.86	3.15
100-52-7	Benzaldehyde	15.05				1.22							2.06	1.29
55012-32-3	Isopropyl benzaldehyde	18.91			2.39									

S1c)

CAS	Aldehydes	Retention time (mins)	25	26	27	28	29	30	31	32	33	34	35	36	37	38
75-07-0	Acetaldehyde	2.16	22.78	122.52	77.79	54.3	35.32	42.75	81.05		33.97	24.51		43.3	12.15	13.02
123-38-6	Propionaldehyde	2.43				5.81			0.97	1.7		1.61	1.75	0.54		0.16
123-72-8	Butyraldehyde	3.10		3.88					8.65	3.36	3.39		4.7	3.64	2.79	3.58
66-25-1	Hexanaldehyde	6.76	15.61		6.98					5.35	5.24		16.45			
100-52-7	Benzaldehyde	15.05				1.92										
55012-32-3	Isopropyl benzaldehyde	18.91														

Tables S1a, S1b, and S1c, Aldehydes found in unmodified stool samples for all participants with retention time and chromatographic peak area.

S2a)														
**CAS**	**Alcohols**	**Retention time (mins)**	**1**	**2**	**3**	**4**	**5**	**6**	**7**	**8**	**9**	**10**	**11**	**12**
64-17-5	Ethanol	3.91	54.64	163.04	244.98	113.15	95.58	129.81	88.27	74.59	61.08	150.72	291.37	925.93
78-92-2	2-Butanol	5.64	22.04		19.74	21.55	29.81	24.51	16.18		14.08	44.64	16.93	12.31
71-23-8	1-Propanol	5.92			87.79				44.53	132.26				
78-83-1	2-Methyl-1-propanol	7.27	11.68	13.1	25.51	24.08	41.83	21.15	14.48	22.89	7.71	15.85	37.54	
598-75-4	3-Methyl -2-butanol	7.80			4.64					4.87				
6032-29-7	2-Pentanol	7.83				7.2			3.1					3.97
71-36-3	1-Butanol	8.29	5.44		55.42	72.25	45.08	21.47	21.97	208.89	6.06		100.22	466.05
123-51-3	3-Methyl -1-butanol	9.51	18.33		13.04	32.73	43.3	24.12	11.86	23.5	20.74	21.16	43.48	17.77
108-11-2	4-Methyl -2-pentanol	9.78												
71-41-0	1-Pentanol	10.35	10		16.52	28.77	36.72	11.49	13.57	56.66	5.03	9.53	19.39	78.22
626-89-1	4-Methyl -1-pentanol	11.55	3.49	3.08	7.38	9.12			7.25	9.5			1.94	
543-49-7	2-Heptanol	11.59												
2313-61-3	4-Methyl -2-hexanol	11.66					3.38							
111-27-3	1-Hexanol	12.24				41.42	49.82			41.96		6.53	2.81	123.3
123-96-6	2-Octanol	13.40					4.94							
3391-86-4	1-Octen-3-ol	13.90	14.42	15.78	6.37	11.98	2.03	1.69						
111-70-6	1-Heptanol	13.99				2.78	15.37							
104-76-7	2-Ethyl-1-hexanol	14.49												
111-87-5	1-Octanol	15.64					4.92							
15356-70-4	Cyclohexanol, 5methyl-2-(1-methylethyl)-,(1a,2b,5a)	16.90						8.01						

S2b)														
**CAS**	**Alcohols**	**Retention time (mins)**	**13**	**14**	**15**	**16**	**17**	**18**	**19**	**20**	**21**	**22**	**23**	**24**
64-17-5	Ethanol	3.91	491.15	113.67	156.52		12.27	253.7	339.14	254.14	139.93	137.65	58.64	151.62
78-92-2	2-Butanol	5.64	13.83	37.33	9.49	9.61	29.76	26.52	7.75	11.86	7.83	64.06	17.84	11.53
71-23-8	1-Propanol	5.92						22.39						7.88
78-83-1	1-Propanol,	7.27	11.54	9.14	13.78	5.55	11.31	54.5	86.4	20.77	10.26	27.88	11.98	44.08
598-75-4	3-Methyl -2-butanol	7.80	1.27				4.44	2.52	1.24					4.87
6032-29-7	2-Pentanol	7.83		11.2					2.35	4.69	1.98			
71-36-3	1-Butanol	8.29	61.48	12.91		55.51	12.25	1.68	132.28	147.5	46.15	28.17	8.71	72.26
123-51-3	3-Methyl -1-butanol	9.51			3.26	6.26	20.27	34.86	32.49	23.46	14.55	18.03	22.28	53.67
108-11-2	4-Methyl -2-pentanol	9.78		5.79										
71-41-0	1-Pentanol	10.35	10.61	6.63	6.33	10.66			29.39					
626-89-1	4-Methyl-1-pentanol	11.55	4.35			3.01		6.93				0.94		2
543-49-7	2-Heptanol	11.59												
2313-61-3	4-Methyl -2-hexanol	11.66												0.85
111-27-3	1-Hexanol	12.24	8.31	5.9	2.69	11.12	3.43	3.82	1.83	2.22	5.53	0.87	1.42	1.34
123-96-6	2-Octanol	13.40	0.6	1.26		0.35								
3391-86-4	1-Octen-3--ol	13.90							0.36					
111-70-6	1-Heptanol	13.99		1.57							7.49	20.46		
104-76-7	2-Ethyl -1-hexanol	14.49												
111-87-5	1-Octanol	15.64												
15356-70-4	Cyclohexanol, 5methyl-2-(1-methylethyl)-,(1a,2b,5a)	16.90												

S2c)																
CAS	Alcohols	Retention time (mins)	25	26	27	28	29	30	31	32	33	34	35	36	37	38
64-17-5	Ethanol	3.91		200.44	96.72	422.76	292.36	159.97	100.69	1052.5	165.17	1479.78	307.18	286.75	54.48	51.63
78-92-2	2-Butanol	5.64	33.7	16.34			9.34		25.22	20.05	4.85		13.7	10.18		
71-23-8	1-Propanol	5.92				320.32										
78-83-1	2-Methyl-1-propanol,	7.27	27.48	37.51	9.11	4.46	41.82	5.77	73.17	19.88	5.75	17.65	8.98	11.93	3.77	9.47
598-75-4	3-Methyl -2-butanol	7.80	28.73						4.95		1.06					
6032-29-7	2-Pentanol	7.83		2.17		47.17										
71-36-3	1-Butanol	8.29	1106.38	40.2	9.3	5.03	20.55	6.73	116.09	307.81	20.58	481.3	49.07	84.58	11.69	13.62
123-51-3	3-Methyl -1-butanol	9.51	30.42	41.5	8.64	15.3	17.23	13.3	73.16	12.06	12.74	8.64	10.49	10.18	3.57	3.47
108-11-2	4-Methyl -2-pentanol	9.78	4.49			24.19								2.18		
71-41-0	1-Pentanol	10.35	23.17			6.24	9.65	4.58	32.7	30.36	0.8	49.63	10.92	9.57	4.3	5.22
626-89-1	4-Methyl -1-pentanol	11.55	15.49	0.73	3.68	0.87		1.49							0.59	
543-49-7	2-Heptanol	11.59					2.07									
2313-61-3	4-Methyl -2-hexanol	11.66			0.46											
111-27-3	1-Hexanol	12.24		9.8		1.88	6.26		26.29	2.55	0.9	1.77				4.38
123-96-6	2-Octanol	13.40				5.06					0.74					
3391-86-4	1-Octen-3--ol	13.90	50.03	3.05	0.73											
111-70-6	1-Heptanol	13.99			2.31											
104-76-7	2-Ethyl -1-hexanol	14.49						5.39								
111-87-5	1-Octanol	15.64		0.64												
15356-70-4	Cyclohexanol, 5methyl-2-(1-methylethyl)-,(1a,2b,5a)	16.90		6.06												

Tables S2a, S2b, and S2c, Alcohols found in unmodified stool samples for all participants with retention time and chromatographic peak area.

S3a)															
CAS	Esters and thioesters	Retention time (mins)	1	2	3	4	5	6	7	8	9	10	11	12
79-20-9	Ethanoic acid, methyl ester	2.69	107.77	101.23	117.83	136.73	104.72	206.31	107.89	177.06	88.00	73.82	104.92	
141-78-6	Ethanoic acid ethyl ester	3.21			13.13			20.11	24.31	17.81			11.33	483.39
105-37-3	Propanoic acid ethyl ester	4.28			18.47			31.27	38.51	26.72			7.5	368.55
97-62-1	2-Methylpropanoic acid ethyl ester	4.44			4.85							1.4		36.65
109-60-4	Ethanoic acid propyl ester	4.60												380.99
623-42-7	Butanoic acid methyl ester	4.79	11.88	9.6	133.9	48.71	10.79	287.95	252.51	268.02		7.24	34	776.39
868-57-5	2-Methylbutanoic acid methyl ester	5.28			12.78			14.28	16.27	15.87				
556-24-1	3-Methylbutanoic acid methyl ester	5.49								20.05				13.51
105-54-4	Butanoic acid ethyl ester	5.87												
106-36-5	Propanoic acid propyl ester	6.04							18.72	66.2				269.37
7452-79-1	2-Methylbutanoic acid ethyl ester	6.21			4.19	7.57		11.56	10.9					
123-86-4	Ethanoic acid butyl ester	6.61								36.17				214.65
624-24-8	Pentanoic acid methyl ester	6.87			20.52	11.51	4.83	52.98	41.21	62.74			13.26	106.62
105-66-8	Butanoic acid propyl ester	7.64			11.58	3.69	4.03	20.92	32.52	34.72			4.29	1205.79
539-82-2	Pentanoic acid ethyl ester	7.92			6.98			14.37	19.25	17.26				425.37
590-01-2	Propanoic acid butyl ester	8.06	77.7		13.23	20.74	16.6	10.96	31.24	50.47	25.32	56.09	1.97	205.3
97-87-0	Propanoic acid 2-methylbutyl ester	8.18												10
539-90-2	Butanoic acid 2-methylpropyl ester	8.45						10.19	9.16	5.29		30.82		20.77
628-63-7	Aethanoic acid pentyl ester	8.68												
123-92-2	Ethanoic acid 3-methylbutanyl ester	8.75								6.02		4.00		27.47
106-70-7	Hexanoic acid methyl ester	9.01				15.44	6.28		21.85	25.74		0.88		293.84
109-21-7	Butanoic acid butyl ester	9.68			5.45	3.25		8.18	7.27	13.29			2.07	877.59
141-06-0	Pentanoic acid propyl ester	9.73							7.66	10.11				
123-66-0	Hexanoic acid ethyl ester	9.99							4.93	10.3				1089.71
624-54-4	Propanoic acid pentyl ester	10.12								4.53				
109-19-3	3 -Methylbutanoic acid butyl ester	10.29								7.41				
106-27-4	Butanoic acid 3-methylbutyl ester	10.60												14.59
142-92-7	Ethanoic acid hexyl ester	10.73												38.28
106-73-0	Heptanoic acid methyl ester	11.02				7.86			4.03	5.8				20.31
591-68-4	Pentanoic acid butyl ester	11.50												
5870-93-9	Butanoic acid heptyl ester	11.51												
626-77-7	Hexanoic acid propyl ester	11.63												438.07
106-30-9	Heptanoic acid ethyl ester	11.88												
4630-82-4	Cyclohexane carboxylic acid methyl ester	12.71								21.93				12.22
111-11-5	Octanoic acid methyl ester	12.82												
626-82-4	Hexanoic acid butyl ester	13.30												149.2

S3b)														
**CAS**	**Esters and thioesters**	**Retention time (mins)**	**13**	**14**	**15**	**16**	**17**	**18**	**19**	**20**	**21**	**22**	**23**	**24**
79-20-9	Ethanoic acid methyl ester	2.69	118.44	130	72.01	118.43	124.64	86.24	109.89	155.81	64.69	103.66	54.19	53.44
141-78-6	Ethanoic acid ethyl ester	3.21	124.04	2.6		138.53		23.95		15.17				
105-37-3	Propanoic acid ethyl ester	4.28	105.99		7.63	152.64	7.6	50.66		13	4.86			9.48
97-62-1	2-Methylpropanoic acid ethyl ester	4.44	22.33			66.71		30.57		4.51	38.36	16.98		2.35
109-60-4	Ethanoic acid propyl ester	4.60	66.09			56.77	223.64			70.46			44.68	5.2
623-42-7	Butanoic acid methyl ester	4.79	104.48	25.53	11.77	248.03	40.94	17.27	16.92		21.62	45.44	7.9	8.67
868-57-5	2-Methylbutanoic acid methyl ester	5.28	3.77			23.62		23.76		2.73				1.7
556-24-1	3-Methylbutanoic acid methyl ester	5.49	11.54		16.09	68.49	37.21	27.56	8.34	8.26	11.34		64.57	26.41
105-54-4	Butanoic acid ethyl ester	5.87	519.8		171.54	630.87		426.27	463.79	141.16		120.9		
106-36-5	Propanoic acid propyl ester	6.04	22.66			15.18								
7452-79-1	2-Methylbutanoic acid ethyl ester	6.21	9.82	4.8		47.99		22.48	6.87	3.74	1.58			2.78
123-86-4	Ethanoic acid butyl ester	6.61	14.5		0.93	22.03				6.2				
624-24-8	Pentanoic acid methyl ester	6.87	18.01	5.43	4.84	41.96	21.58	2						
105-66-8	Butanoic acid propyl ester	7.64	49.85		2.4	20.98		50.3		8.19	7.79		2.32	
539-82-2	Pentanoic acid ethyl ester	7.92	58.21		2.49	93.66		51.21	1.55	2.87	4.63		89.42	3.57
590-01-2	Propanoic acid butyl ester	8.06	9.91		16.35	19.29		2.53			5.07	7.68	16.4	
97-87-0	Propanoic acid 2-methyl butyl ester	8.18			11.58		11.47	8.79						
539-90-2	Butanoic acid 2-methylpropyl ester	8.45		4.81	1.1	9.24		57.99						
628-63-7	Aethanoic acid pentyl ester	8.68												
123-92-2	Ethanoic acid 3-methyl butanyl ester	8.75	1.44			3.03	18.62	1.93		0.71	4.23	1.1	4.04	1.47
106-70-7	Hexanoic acid methyl ester	9.01	14.52	5.54	1.43	2.49			4.32	7.74		1.36		22.93
109-21-7	Butanoic acid butyl ester	9.68	18.3			9.62	2.32	17.78	2.56	4.17		1.45		2.92
141-06-0	Pentanoic acid propyl ester	9.73	8				1.45	16.69						1.67
123-66-0	Hexanoic acid ethyl ester	9.99	42.59			70.59	0.68	1.74			1.77		0.79	
624-54-4	Propanoic acid pentyl ester	10.12	1.22	38.46	1.46			4.07					7.56	
109-19-3	3- Methylbutanoic acid butyl ester	10.29					10.09	18.27		21.9	10.56	10.36	4.38	26.15
106-27-4	Butanoic acid 3-methylbutyl ester	10.60	1.39				3.01						6.72	
142-92-7	Ethanoic acid hexyl ester	10.73	0.92			1.55	0.99	0.98			1.11	0.91	2.4	
106-73-0	Heptanoic acid methyl ester	11.02	3.2		2.4	4.47								
591-68-4	Pentanoic acid butyl ester	11.50												
5870-93-9	Butanoic acid heptyl ester	11.51												
626-77-7	Hexanoic acid propyl ester	11.63	5.72	4.29		2.59	0.73							
106-30-9	Heptanoic acid ethyl ester	11.88	3.78			2.66	30.09	17.84	30.75	20.37	6.8	9.82	50.34	71.99
4630-82-4	Cyclohexane carboxylic acid methyl ester	12.71					1.02						0.42	
111-11-5	Octanoic acid methyl ester	12.82												
626-82-4	Hexanoic acid, butyl ester	13.30	1.35		2.15	1.28	0.4							0.39

S3c)																
**CAS**	**Esters and thioesters**	**Retention time (mins)**	**25**	**26**	**27**	**28**	**29**	**30**	**31**	**32**	**33**	**34**	**35**	**36**	**37**	**38**
79-20-9	Ethanoic acid, methyl ester	2.69	506.42	216.74	75.02	120.83	79.75	129.05	129.68	242.88	203.39	556.94	4.12	69.11	11.85	18.14
141-78-6	Ethanoic acid ethyl ester	3.21		13.79		93.82	2.96		11.35	229.27	26.4		2.42	2.02	0.7	0.84
105-37-3	Propanoic acid ethyl ester	4.28				449.67			11.76	81.22	48.23	833.25	46.61	3.07	2.89	1.7
97-62-1	2-Methyl propanoic acid ethyl ester	4.44	49.1			15.19			78.57	5.26						
109-60-4	Ethanoic acid propyl ester	4.60				17.25				80.21	54.21	482.53			5.19	
623-42-7	Butanoic acid methyl ester	4.79		8.32	5.46	84.59	38.56	11.42	204	597.33	335.208	2314.9	17.12	58.6	9.25	7.53
868-57-5	2-Methyl butanoic acid methyl ester	5.28				31.96			12.26		4.83			1.25	51.36	
556-24-1	3-Methyl butanoic acid methyl ester	5.49	13.25	19.77								16.16				
105-54-4	Butanoic acid ethyl ester	5.87		164.15						1075.92	287.35			198.35		
106-36-5	Propanoic acid propyl ester	6.04		2.8							68.87					
7452-79-1	2-Methyl butanoic acid ethyl ester	6.21	472.99		2.5	19.33			2.12		4.92					
123-86-4	Ethanoic acid butyl ester	6.61	435.4			4.98				30.89		277.58				
624-24-8	Pentanoic acid methyl ester	6.87	67.82	3.09	1.7	5.6	8.43	5.75	29.36	61.61	24.68	332.4	49.45	11.04		
105-66-8	Butanoic acid propyl ester	7.64	1043.23		1.46	25.92	1.12		21.4	94.32	39.03	136.07	1.68	4.41	2.38	
539-82-2	Pentanoic acid ethyl ester	7.92		6.21	1.55	3.35			2.91	59.3	2.94	788.31		1.89	1.04	
590-01-2	Propanoic acid butyl ester	8.06	361.95	15.37		12.49			7.61			208.25		2.07	0.95	0.74
97-87-0	Propanoic acid 2-methylbutyl ester	8.18				4.88										
539-90-2	Butanoic acid 2-methylpropyl ester	8.45			0.85				7.83			67.33				
628-63-7	Aethanoic acid pentyl ester	8.68							3.24			19.12				
123-92-2	Ethanoic acid 3-methyl butanyl ester	8.75	8.39		3.17	2.91										
106-70-7	Hexanoic acid methyl ester	9.01				2.98	7.81	1.33	29.21	1.24		5.36				3.1
109-21-7	Butanoic acid butyl ester	9.68		2.55		17.29			18.27	55.87	4.81	715.41		2.03	1.25	0.78
141-06-0	Pentanoic acid propyl ester	9.73			0.43											
123-66-0	Hexanoic acid ethyl ester	9.99	8.2		0.47				2.31			19.14			0.46	0.44
624-54-4	Propanoic acid pentyl ester	10.12	2.53			0.47			1.18		3.59	9.77				
109-19-3	3- Methyl butanoic acid butyl ester	10.29	14.78	18.36	4.82	2.53					4.57				1.25	
106-27-4	Butanoic acid 3-methylbutyl ester	10.60							4.9			7.82				
142-92-7	Ethanoic acid hexyl ester	10.73	3.57			14.43			1.55			2.76				
106-73-0	Heptanoic acid methyl ester	11.02							8.64							0.73
591-68-4	Pentanoic acid butyl ester	11.50										78.93				
5870-93-9	Butanoic acid heptyl ester	11.51							10.84	7.5						
626-77-7	Hexanoic acid propyl ester	11.63	3.03	2.14												
106-30-9	Heptanoic acid ethyl ester	11.88	57.61	27.71	10.9	1.23							1.04			
4630-82-4	Cyclohexane carboxylic acid methyl ester	12.71	3.29			1.29					12.16					
111-11-5	Octanoic acid methyl ester	12.82						2.21								
626-82-4	Hexanoic acid, butyl ester	13.30			0.2				6.12			2.65				

Tables S3a, S3b, and S3c, Esters and thioesters found in unmodifed stool samples for all participants with retention time and chromatographic peak area.

S4a)

CAS	Ketones	Retention time (mins)	1	2	3	4	5	6	7	8	9	10	11	12
67-64-1	Acetone	2.61	148.03	187.16	184.56	179.32	193.52	404.75	363.6	146.87	162.02	170.04	202.18	210.3
78-93-3	2-Butanone	3.37		546.72										
563-80-4	3-Methyl 2-butanone	4.56												
107-87-9	2-Pentanone	4.61		37.44		198.34	58.79	141.58	108.36	117.22		29.05	42.67	
431-03-8	2,3-Butanedione	4.61			47.06									
108-10-1	Methyl isobutyl ketone	5.19				27.36								
591-78-6	2-Hexanone	5.21						12.93						
565-61-7	3-Methyl 2-pentanone	5.37				12.83					5.82			
600-14-6	2,3- Pentanedione	6.33											19.55	
105-42-0	4-Methyl 2-hexanone	8.84												
110-43-0	2-Heptanone	8.91			3.94	17.39	26.59	4.68	8.23	1.96	4.03	3.86	2.35	
110-93-0	6-Methyl -5-hepten-2-one	11.94	68.91	98.65	43.13	37.69	15.41	101.06	9.14	4.33	52.48	12.54	43.31	100.71

S4b)

CAS	Ketones	Retention time (mins)	13	14	15	16	17	18	19	20	21	22	23	24
67-64-1	Acetone	2.61	206.65	340.64	25.04	55.52	533.55	186.84	109.9	95.74	154.69	148.61	466.96	151.9
78-93-3	2-Butanone	3.37												
563-80-4	3-Methyl 2-butanone	4.56												
107-87-9	2-Pentanone	4.61		557.96				27.73	26.39					
431-03-8	2,3-Butanedione	4.61												
108-10-1	Methyl isobutyl ketone	5.19	7.02	20.15					6.1					
591-78-6	2-Hexanone	5.21					3.29			2.65	3.4	5.38		
565-61-7	3-Methyl -2-pentanone	5.37		15.59					9.35		5.04	6.85		
600-14-6	2,3- Pentanedione	6.33	7.47		10.75			6.9		11.51	2.21		5.59	
105-42-0	4-Methyl- 2-hexanone	8.84												
110-43-0	2-Heptanone	8.91	5.63	23.09		2.53	11.61		0.98	1.59	2.83		3.91	
110-93-0	6-Methyl -5-hepten-2-one	11.94	0.78	13	7.47	3.2		1.57	0.86					

S4c)

CAS	Ketones	Retention time (mins)	25	26	27	28	29	30	31	32	33	34	35	36	37	38
67-64-1	Acetone	2.61	211.47	240.61	78.31	43.06	73.78	356.22	286.22	103.38	56.55	110.4	340.88	185.94	37.79	20.18
78-93-3	2-Butanone	3.37														
563-80-4	3-Methyl -2-butanone	4.56														2.89
107-87-9	2-Pentanone	4.61		48.74	85.84											
431-03-8	2,3-Butanedione	4.61					6.95	17.86								
108-10-1	Methyl isobutyl ketone	5.19			2.54	4.53					5.31					
591-78-6	2-Hexanone	5.21	14.19													
565-61-7	3-Methyl -2-pentanone	5.37	24.08											1.46		
600-14-6	2,3,- Pentanedione	6.33	65.04	9.48					2.04		2.22					
105-42-0	4-Methyl -2-hexanone	8.84					1.58	0.99	3.91	1.47	2.69		1			
110-43-0	2-Heptanone	8.91	15.36	2.32	0.99											
110-93-0	6-Methyl -5-hepten-2-one	11.94	3.27				1.54			1.81		2.17				

Tables S4a, S4b, and S4c, Ketones found in unmodifiied stool samples for all participants with retention time and chromatographic peak area.

S5a)

CAS	Acids	Retention time (mins)	1	2	3	4	5	6	7	8	9	10	11	12
64-19-7	Ethanoic acid	14.11	36.5	49.85	39.26	84.42	29.61	27.56	29.65	184.72	83.28	60.75	56.5	61.11
79-09-4	Propanoic acid	15.42								116.3	44.51	20.4	13.88	27.17
79-31-2	2-Methylpropanoic acid	15.93	1.99	1.48	2.26	2.96	1.84	2.46	9.25	63.54	21.57	10.37	6.5	12.15
107-92-6	Butanoic acid	16.82	17.25	13.78	18.45	46.21	18.42	12.8	25.93	322.75	133.6	60.27	44.23	78.16
503-74-2	3-Methylbutanoic acid	17.33	8.63	10.39	11.6	10.3	10.31	10.46	19.8	110.89	39.97	18.27	17.16	32.86
109-52-4	Pentanoic acid	18.40	2.19		4.54			4.04	14.06	89.51		12.82	13.84	20.04
142-62-1	Hexanoic acid	19.77												
111-14-8	Heptanoic acid	21.23												3.71
98-89-5	Cyclohexanecarboxylic acid	22.89								9.94				
65-85-0	Benzoic acid	27.00				25.97								

S5b)

CAS	Acids	Retention time (mins)	13	14	15	16	17	18	19	20	21	22	23	24
64-19-7	Ethanoic acid	14.11	56.46	120.09	5.05	68.57		52.97	20.96	15.61			31.51	20.94
79-09-4	Propanoic acid	15.42	20.73	34.6	8.38		8.3	15.66	4.93	2.89	1.61	3.98	3.22	3.44
79-31-2	2-Methylpropanoic acid	15.93	9.67	11.61	3.24	11.81	6.15	12.07	3.41	2.14	0.75	2.29	1.26	2.11
107-92-6	Butanoic acid	16.82	71.31	99.34	29.87	85.42	40.99	57.1	16.49	9.48	4.36	12.36	10.47	13.74
503-74-2	3-Methylbutanoic acid	17.33	19.9	23.13	7.08	20.46	12.43	22.91	6.66	4.53		4.43	3	4
109-52-4	Pentanoic acid	18.40	15.51	12.86	2.96	11.14	6.92	11.14	4.74	1.93		2.47	1.86	
142-62-1	Hexanoic acid	19.77												
111-14-8	Heptanoic acid	21.23					0.91							
98-89-5	Cyclohexanecarboxylic acid	22.89												
65-85-0	Benzoic acid	27.00												

S5c)																
CAS	Acids	Retention time (mins)	25	26	27	28	29	30	31	32	33	34	35	36	37	38
64-19-7	Ethanoic acid	14.11		53.75		10.72	20.82	26.06	230.18	101.34	8.05	179.82	87.46	24.03	12.41	14.98
79-09-4	Propanoic acid	15.42		12.27	14.99	3.32		5.56	94.61	30.23	30.4	74.44	32.75	5.37	2.67	3.09
79-31-2	2-Methylpropanoic acid	15.93	92.22	3.49				2.81	71.8	21.83	16.47	30.53	15.91	2.84	2.24	1.59
107-92-6	Butanoic acid	16.82	637.09	48.91	18.02	11.88	13.49	23	364.97	116.71	102.47	274.48	135.55	25.23	12.58	16.38
503-74-2	3-Methylbutanoic acid	17.33	162.97	9.08				5.74	133.1	45.8	30.86	41.62	22.97	5.53	2.56	5.54
109-52-4	Pentanoic acid	18.40		5.77			5.12	3.35	64.32	28.85	16.77	37.22	22.59	7.38	2.75	2.3
142-62-1	Hexanoic acid	19.77							2.16	12.38	6.69	17.93	7.8		1.72	
111-14-8	Heptanoic acid	21.23			2.66				4.32							
98-89-5	Cyclohexanecarboxylic acid	22.89			1.43											
65-85-0	Benzoic acid	27.00														

Tables S5a, S5b, and S5c, Acid found in unmodified stool samples for all participants with retention time and chromatographic peak area.

S6a)														
**CAS**	**Nitrogen containing compounds**	**Retention time (mins)**	**1**	**2**	**3**	**4**	**5**	**6**	**7**	**8**	**9**	**10**	**11**	**12**
75-05-8	Acetonitrile	5.10	5.42	13.59	9.33						10.6	9.69	17.7	
7149-26-0	1,6-Octadien-3-ol, 3,7-dimethyl-2-aminobenzoate	15.63		12.59		1.18								
120-72-9	Indole	26.78	22.53	26.36	14.06	12.37	14.87	13.82	39.7	3.13				
83-34-1	3-Methylindole	27.27	16.84	27.74	19.94	12.67	20.08	7.09		0.5				

S6b)														
**CAS**	**Nitrogen containing compounds**	**Retention time (mins)**	**13**	**14**	**15**	**16**	**17**	**18**	**19**	**20**	**21**	**22**	**23**	**24**
75-05-8	Acetonitrile	5.10	5.01		12.58		3.55		3.85	3.7	5.9	4.44	9.5	2.89
7149-26-0	1,6-Octadien-3-ol, 3,7-dimethyl-2-aminobenzoate	15.63		1.82		1.98								
120-72-9	Indole	26.78					17.61	40.36	18.46	6.19	5.89	6.72	5.88	8.99
83-34-1	3-Methylindole	27.27					11.87	13.09	9.63	3.35	15.19	3.98	7.81	7.62

S6c)																
**CAS**	**Nitrogen containing compounds**	**Retention time (mins)**	**25**	**26**	**27**	**28**	**29**	**30**	**31**	**32**	**33**	**34**	**35**	**36**	**37**	**38**
75-05-8	Acetonitrile	5.10		12.72	4.43	3.94	4.44	5.72	6.37	3.06			7.52	2		
7149-26-0	1,6-Octadien-3-ol, 3,7-dimethyl-2-aminobenzoate	15.63			1.68											
120-72-9	Indole	26.78	20.56	31.73	7.11	12.9	71	14.5	21.78	19.25		13.5	9.8	18.1		
83-34-1	3-Methylindole	27.27	10.89	12.14	3.77	3.14	3.16	6.62	5.7	12.42						

Tables S6a, S6b, and S6c, Nitrogen containing compounds found in unmodifed stool samples for all participants with retention time and chromatographic peak area.

S7a)

CAS	Sulpher compounds	Retention time (mins)	1	2	3	4	5	6	7	8	9	10	11	12
75-18-3	Dimethyl sulphide	2.30	203.39	56.77	16.53		23.15	45.02	223.11	203.39	374.49	42.98	281.73	106.79
624-92-0	Dimethyl disuphide	6.52	231.45	325.68	86.61	197.58	195.1	259.84	213.6	178.59	200.21	168.97	834.04	188.52
5925-75-7	S-Methyl propanethioate	7.45	42.57	20.19	6.1	1.22	9.1	13.4		2	0.79	36.37		
23747-45-7	S-Methyl 3-methylbutanethioate	9.69												
2179-60-4	Methyl propyl disulphide	9.87	3.4		1.94		3.87			0.93			2.59	
1618-26-4	2,4-Dithiapentane	10.60											7.07	
57-06-7	Allyl isothiocyanate	12.32				13.45					6.97		5.42	
3658-80-8	Dimethyl trisulphide	12.65	9.41	17.77	4.59	9.3	9.92	9.62	14.5		3.81	2.17	52.99	

S7b)

CAS	Sulpher compounds	Retention time (mins)	13	14	15	16	17	18	19	20	21	22	23	24
75-18-3	Dimethyl sulphide	2.30	16.7	17.12		55.47	24.21	238.77	37.98	44.42	19.91	165.05		32.7
624-92-0	Dimethyl disulphide	6.52	296.83	516.85	224.59	362.51	166.39	566.67	484.93	200.35	212.21	237.7	200.09	604.71
5925-75-7	S-Methyl propanethioate	7.45			5.73	10.85							13.79	
23747-45-7	S-Methyl 3-methylbutanethioate	9.69												
2179-60-4	Methyl propyl disulphide	9.87	1.58	2.99										
1618-26-4	2,4-Dithiapentane	10.60							0.54					
57-06-7	Allyl isothiocyanate	12.32	0.55	2.01		0.7		2.3	1.8					0.89
3658-80-8	Dimethyl trisulfide	12.65	20.62	20.93	3	15.81	5.99	39.63	24.67	1.82	4.67	10.05	2.82	2.69

S7c)

CAS	Sulpher compounds	Retention time (mins)	25	26	27	28	29	30	31	32	33	34	35	36	37	38
75-18-3	Dimethyl sulphide	2.30	8.79	282.86	20.75	28.7	57.14	81.34	34.74	137.39		46.26	65.97	129.46	350.25	18.64
624-92-0	Dimethyl disuphide	6.52	491.65	412.1	23.49	197.74	488.56	183.41	762.2	267.79	117.79	136.73	464.7	496.07	196.26	71.58
5925-75-7	S-Methyl propanethioate	7.45					1.45									
23747-45-7	S-Methyl 3-methylbutanethioate	9.69					3.57		18.2	2.08			1.8	1.6		
2179-60-4	Methyl propyl disulphide	9.87		3.02			4.78	1.34	1.77		0.84				0.51	
1618-26-4	2,4-Dithiapentane	10.60		1.13												
57-06-7	Allyl isothiocyanate	12.32	13.61	1.24		1.23										
3658-80-8	Dimethyl trisulfide	12.65	63.43	19.61	13.68	13.54	43.15	4.32	62.39	27.27		2.94	36.14	39.72	11.54	4.25

Tables S7a, S7b, and S7c, Sulpher containing compounds found in unmodifed stool samples for all participants with retention time and chromatographic peak area.

S8a)														
CAS	Miscellaneous	Retention time (mins)	1	2	3	4	5	6	7	8	9	10	11	12
75-09-2	Dichloromethane	3.78	82.96	144.14	46.62						92.1			
1073-91-2	1,2,4,5- Tetroxane, 3,3,6,6-tetramethyl	4.63	18.86								13.29			

S8b)														
CAS	Miscellaneous	Retention time (mins)	13	14	15	16	17	18	19	20	21	22	23	24
75-09-2	Dichloromethane	3.78	73.89		77.16		227.47	92.56	68.45	151.42		117.81	148.9	95.33
1073-91-2	1,2,4,5- Tetroxane, 3,3,6,6-tetramethyl	4.63			33.38									

S8c)																
CAS	Miscellaneous	Retention time (mins)	25	26	27	28	3029	30	31	32	33	34	35	36	37	38
75-09-2	Dichloromethane	3.78	97.01	92.4	107.9	55.36	44.6	130.81	83.48		44.97		95.41	84.58	31.43	20.89
1073-91-2	1,2,4,5- Tetroxane, 3,3,6,6-tetramethyl	4.63												22.79		

Tables S8a, S8b, and S8c, Uncategorised compound found in unmodified stool samples for all participants with retention time and chromatographic peak area.

S9a)														
CAS	Aromatic compounds	Retention time (mins)	1	2	3	4	5	6	7	8	9	10	11	12
7149-26-0	1,6-Octadien-3-ol, 3,7-dimethyl-2-aminobenzoate	15.63		12.59		1.18								
140-67-0	Estragole	17.32												
108-95-2	Phenol	21.84	10.65	12.26	20.53	21.72	9.4	10.36	13.1	16.14	11.66	10.85	14.11	12.48
106-44-5	4-Methylphenol	22.76	49.39	76.34	67.8	40.56	81.97	97.82	79.73	52.38	63.66	37.6	61.66	72.45
120-72-9	Indole	26.78	22.53	26.36	14.06	12.37	14.87	13.82	39.7	3.13				
83-34-1	3-Methyl indole	27.27	16.84	27.74	19.94	12.67	20.08	7.09		0.5				

S9b)														
CAS	Aromatic compounds	Retention time (mins)	13	14	15	16	17	18	19	20	21	22	23	24
7149-26-0	1,6-Octadien-3-ol, 3,7-dimethyl-,2-aminobenzoate	15.63		1.82		1.98								
140-67-0	Estragole	17.32				4.39					2.38			
108-95-2	Phenol	21.84	5.33	5.27	4.94	5.18	4.2	5.79	6.03	3.94	3.39		15.67	10.76
106-44-5	4-Methylphenol	22.76	84.54	97.97	112.53	55.98	47.26	144.16	102.33	72.75	65.4	97.56	91.74	114.85
120-72-9	Indole	26.78					17.61	40.36	18.46	6.19	5.89	6.72	5.88	8.99

83-34-1	3-Methylindole	27.27					11.87	13.09	9.63	3.35	15.19	3.98	7.81	7.62

S9c)

CAS	Aromatic compounds	Retention time (mins)	25	26	27	28	29	30	31	32	33	34	35	36	37	38
7149-26-0	1,6-Octadien-3-ol, 3,7-dimethyl-,2-aminobenzoate	15.63			1.68											
140-67-0	Estragole	17.32														
108-95-2	Phenol	21.84	22.65	4.8	8.49	7.39	12.26	8.46	5.98	5.92	14.57	11.32	4.98	4.94	2.12	1.72
106-44-5	4-Methylphenol	22.76	179.67	250.49	60.76	42.3	39.49		155.92	54.74	15.67	13.52	32.45	22.78	5.48	3.89
120-72-9	Indole	26.78	20.56	31.73	7.11	12.9	71	14.5	21.78	19.25		13.5	9.8	18.1		
83-34-1	3-Methylindole	27.27	10.89	12.14	3.77	3.14	3.16	6.62	5.7	12.42						

Tables S9a, S9b, and S9c, Aromatic compounds found in unmodified stool samples for all participants with retention time and chromatographic peak area.

S10a)

Siloxanes	Retention time (mins)	1	2	3	4	5	6	7	8	9	10	11	12
Siloxane (1)	4.31				19.99								
Siloxane (2)	13.03		10.44	4.62		5.28	3.64	4.06	8.42	3.48	2.89	3.97	
Siloxane (3)	15.32	4.81	5.16	4.57	5.4	6.49	3.88	5.51		2.03	1.94	2.34	

S10b)

Siloxanes	Retention time (mins)	13	14	15	16	17	18	19	20	21	22	23	24
Siloxane (1)	4.31												
Siloxane (2)	13.03	1.48	2.94		4.09	1.62	2.61	2.43	0.92		2.89	1.97	1.31
Siloxane (3)	15.32				1.08								

S10c)

Siloxanes	Retention time (mins)	25	26	27	28	29	30	31	32	33	34	35	36	37	38
Siloxane (1)	4.31														
Siloxane (2)	13.03	20.89	2.66	29.28			1.38	7.13	11.49	7.12	2.57		2.38	1.46	
Siloxane (3)	15.32	18.68			2.87										

Tables S10a, S10b, and S10c, Siloxanes found in unmodified stool samples for all participants with retention time and chromatographic peak area.

S11a)

Terpenes	Retention time (mins)	1	2	3	4	5	6	7	8	9	10	11	12
Terpene (1)	3.12	18.15								11.49	10.61		
Terpene (2)	4.13												
Terpene (3)	5.30												
Terpene (4)	5.52	60.25	187.79	27.85	21.87	44.69	27.29	28.09		18.77	113.72		
Terpene (5)	5.78												
Terpene (6)	6.39					8.39				12.39	4.15		
Terpene (7)	7.13	69.38	35.86	10.72	14.75	134.4	17.7	11.42	7.53	14.13	262.89		16.46
Terpene (8)	7.37												
Terpene (9)	8.16					27.09	4.43	8.42	6.65		4.37	6.97	
Terpene (10)	8.42	13.47	531.5			10.12						1.8	
Terpene (11)	8.47									7			
Terpene (12)	9.16	81.61	15555.7	33.19	13.06	21.47	243.65	26.07	60.62	21.39	79.09	9.22	6.75
Terpene (13)	9.34	6.68				4.89	5.8			4.38	9.65		
Terpene (14)	9.92												
Terpene (15)	10.16	27.03	11.71	22.36	6.54	79.37	5.37	2.21		4.1	114.69	1.24	
Terpene (16)	10.38		14.73										
Terpene (17)	10.63	13.3	42.15	14.48	3.27	32.28	10.11	3.14	1.9	3.27	132.89		
Terpene (18)	10.95		14.7			16.42							
Terpene (19)	13.69		63.44								0.8		
Terpene (20)	13.73			7.17									
Terpene (21)	14.56					2.07							
Terpene (22)	14.73		40.71	0.66		3.45	1.33	0.71			2.58		
Terpene (23)	15.47		4.6	4.52	7.8	3.62	3.41						
Terpene (24)	16.16	3.9											
Terpene (25)	16.35	15.42	27.56	3.8	5.09	27.95	7.87	6.64	1.87	4.79	29.53	3.31	
Terpene (26)	17.99					1.97							
Terpene (27)	18.12									6.66			
Terpene (28)	18.22	1.53											
Terpene (29)	20.71		33.18	7.86						10.91	6.86		
Terpene (29)	23.86		2.27	1.21	3.21				1.1			0.91	
Terpene (30)	24.64				14.18	6.41							

S11b)

Terpenes	Retention time (mins)	13	14	15	16	17	18	19	20	21	22	23	24
Terpene (1)	3.12			34.48									
Terpene (2)	4.13												
Terpene (3)	5.30												
Terpene (4)	5.52		17.42										
Terpene (5)	5.78					106.26			126.39	67.9		97.28	150.86
Terpene (6)	6.39												
Terpene (7)	7.13		60.76	11.9	171.24	30.42						38.37	
Terpene (8)	7.37												
Terpene (9)	8.16	1.37		4.62	9.99	66.92							
Terpene (10)	8.42	3.26		1.99	3.49	4.07							
Terpene (11)	8.47					0.79	13.37		0.49				
Terpene (12)	9.16	6.94		6.44	42.26	36.39	3.51		2.7	28.21		57.74	
Terpene (13)	9.34			2.05	3.63	1.1	0.88		3.74			3.24	2.59
Terpene (14)	9.92												
Terpene (15)	10.16				84.68								
Terpene (16)	10.38					2.15							2.23
Terpene (17)	10.63		21.08	1.22	28.48								
Terpene (18)	10.95		4.67			4.22	2.32	0.95					1.48
Terpene (19)	13.69					39.61						0.39	
Terpene (20)	13.73												
Terpene (21)	14.56									1.1			
Terpene (22)	14.73			1.3	1.07	2.85						1.25	
Terpene (23)	15.47				25.27								
Terpene (24)	16.16												
Terpene (25)	16.35	1.84	3.8	10.77	4.62	3.19	2.81	1.44		0.99			1.28
Terpene (26)	17.99												
Terpene (27)	18.12		3.22	4.81					0.45				
Terpene (28)	18.22		28.78	2.73									2.63
Terpene (29)	20.71												
Terpene (29)	23.86												
Terpene (30)	24.64												

S11c)															
**Terpenes**	**Retention time (mins)**	**25**	**26**	**27**	**28**	**29**	**30**	**31**	**32**	**33**	**34**	**35**	**36**	**37**	**38**
Terpene (1)	3.12														
Terpene (2)	4.13					4.25									
Terpene (3)	5.30						1.57		3.19		14.17		1.02		
Terpene (4)	5.52			7.23		12.62	27.49		21.48	14.43					
Terpene (5)	5.78			47.1			103.61								
Terpene (6)	6.39					5.39	7.25				18.53				
Terpene (7)	7.13	16.09	3.39	2.91	1.52	3.59	4.83		17.83	5.59	2.12		3.23	1.38	
Terpene (8)	7.37						2.65		4.2	3.91	2.65				
Terpene (9)	8.16	16.58				4.72	2.54		45.33	14.67				3.74	
Terpene (10)	8.42					2.84	0.75			2.91				0.76	
Terpene (11)	8.47		8.03						2.01						
Terpene (12)	9.16	53.23	4.56	6.7		10.97	26.35	2.76	23.68	7.94	287.27	1.34	4.17	5.28	0.74
Terpene (13)	9.34	1.2	2			22.72	2.67		2.05	1.38	13.76				
Terpene (14)	9.92					3.84			1.18						
Terpene (15)	10.16					1.67	5.04							0.48	
Terpene (16)	10.38	43.96													
Terpene (17)	10.63					6.12	1.89			2.49					
Terpene (18)	10.95	2.49	2.5		3.15		2.97					2.55			
Terpene (19)	13.69				3.97						0.72				
Terpene (20)	13.73														
Terpene (21)	14.56		2.5	0.77											
Terpene (22)	14.73		1.17												
Terpene (23)	15.47			4.76		7.02									
Terpene (24)	16.16														
Terpene (25)	16.35	3.85	4.7		5.37	4.53	7.08		2.37	1.75			2		
Terpene (26)	17.99														
Terpene (27)	18.12	100.88					1.56								
Terpene (28)	18.22			1.77											
Terpene (29)	20.71		26.1		5.52	13.81	17.34		0.89	1.07					
Terpene (29)	23.86										1.15				
Terpene (30)	24.64														

Tables S11a, S11b, and S11c, Terpenes found in unmodified stool samples for all participants with retention time and chromatographic peak area.

S12a)

Unidentified	Retention time (mins)	1	2	3	4	5	6	7	8	9	10	11	12
Unidentified (1)	3.46		954.93										1149.81
Unidentified (2)	3.81				103.68			111.2	122.12		47.91	73.41	
Unidentified (3)	4.38												
Unidentified (4)	5.19			10.58		13.99							
Unidentified (5)	5.37			10.07		5.87					9.93		
Unidentified (6)	5.84	10.02	116.36	88.28	160.57							114.57	
Unidentified (7)	5.87					97.72	186.13	175.49	144.23		66.06		
Unidentified (8)	6.04												
Unidentified (9)	6.23								12.49				55.16
Unidentified (10)	6.36	3.38	6.95		6.61				0.8				
Unidentified (11)	6.67												
Unidentified (12)	6.92		13.3										
Unidentified (13)	7.60	2.27	6.74										
Unidentified (14)	7.91	5.42									1.72	6.18	
Unidentified (15)	9.58							3.37					
Unidentified (16)	10.43											5.88	
Unidentified (17)	10.53												
Unidentified (18)	11.26	2.03						5.05	2.97			1.67	
Unidentified (19)	12.04											3.82	
Unidentified (20)	12.29												
Unidentified (21)	13.31								2.82				
Unidentified (22)	13.35							1.31					
Unidentified (21)	15.02								5.04				17.57
Unidentified (22)	17.58					1.27							
Unidentified (23)	17.75					5.47					1.82	1.18	
Unidentified (24)	19.86												
Unidentified (25)	20.31	4.41	7.07	3.5		62.59							
Unidentified (26)	20.82								2.73				
Unidentified (27)	21.22						2.19	3.4	6.69	4.54	2.32		
Unidentified (28)	21.56						2.62	2.2	3.18	2.22			
Unidentified (29)	21.99	13.55	7.47										
Unidentified (30)	25.61						8.1						
Unidentified (31)	25.88	12.58	10.95	10.14		5.53							
Unidentified (32)	26.97					18.77	7.7						

S12b)													
**Unidentified**	**Retention time (mins)**	**13**	**14**	**15**	**16**	**17**	**18**	**19**	**20**	**21**	**22**	**23**	**24**
Unidentified (1)	3.46												
Unidentified (2)	3.81		85.19			97.28				151.12			
Unidentified (3)	4.38												
Unidentified (4)	5.19					7.92			4.68		11.4	14.33	
Unidentified (5)	5.37	11.82											
Unidentified (6)	5.84		74.7										
Unidentified (7)	5.87												
Unidentified (8)	6.04												
Unidentified (9)	6.23												
Unidentified (10)	6.36		12.36		2.82								
Unidentified (11)	6.67						9.8	4.9	9.34	4.53		24.08	14.01
Unidentified (12)	6.92												
Unidentified (13)	7.60							5.83					5.34
Unidentified (14)	7.91												
Unidentified (15)	9.58												1.07
Unidentified (16)	10.43	2.11	2.64	6.62	1.89		2.14	3.02	1.47	1.28	1.79	1.83	
Unidentified (17)	10.53						6.09		0.79				1.19
Unidentified (18)	11.26	0.48	1.19		0.97		1.14						
Unidentified (19)	12.04	1.36	0.77		1.58								
Unidentified (20)	12.29												
Unidentified (21)	13.31												
Unidentified (22)	13.35	1.17											
Unidentified (21)	15.02		1.35	3.12			2.41	1.2	0.64		0.8		
Unidentified (22)	17.58												
Unidentified (23)	17.75			2.49									
Unidentified (24)	19.86												
Unidentified (25)	20.31						1.2						
Unidentified (26)	20.82				1.88					23.07	23.97		
Unidentified (27)	21.22	1.47	1.34										
Unidentified (28)	21.56												
Unidentified (29)	21.99												
Unidentified (30)	25.61												
Unidentified (31)	25.88												
Unidentified (32)	26.97											6.64	

S12c)															
**Unidentified**	**Retention time (mins)**	**25**	**26**	**27**	**28**	**29**	**30**	**31**	**32**	**33**	**34**	**35**	**36**	**37**	**38**
Unidentified (1)	3.46														
Unidentified (2)	3.81														
Unidentified (3)	4.38							2.33							
Unidentified (4)	5.19														
Unidentified (5)	5.37			2.76						2.76					
Unidentified (6)	5.84					138.32		206.48				237.85			21.89
Unidentified (7)	5.87														
Unidentified (8)	6.04										7023.54				
Unidentified (9)	6.23														
Unidentified (10)	6.36														
Unidentified (11)	6.67		10.06				2.14	11.77			7.02		2.7		
Unidentified (12)	6.92														
Unidentified (13)	7.60		2.27				4.42								
Unidentified (14)	7.91	365.96				0.86							0.78		
Unidentified (15)	9.58	419.43													
Unidentified (16)	10.43	3.52		5.45								2			
Unidentified (17)	10.53	12.84			1.44				6.49						
Unidentified (18)	11.26	1.41			0.26			3.89							0.5
Unidentified (19)	12.04													0.5	
Unidentified (20)	12.29					1.86									
Unidentified (21)	13.31					0.53									
Unidentified (22)	13.35	13.55				0.62			5.1						
Unidentified (21)	15.02	16.88						3.1							
Unidentified (22)	17.58			9.13		2.08									
Unidentified (23)	17.75			0.51											
Unidentified (24)	19.86														1.22
Unidentified (25)	20.31						3.88								
Unidentified (26)	20.82			8.81											
Unidentified (27)	21.22														
Unidentified (28)	21.56			3.96											

Unidentified (29)	21.99											
Unidentified (30)	25.61	0.31										1.72
Unidentified (31)	25.88	2.02										
Unidentified (32)	26.97		0.51									

Tables S12a, S12b, and S12c, Unidentified compounds found in unmodified stool samples for all participants, with retention time and chromatographic peak area.

CAS	Compound name	RT (mins)	RI exp (KI)	RI lit [A] (F. Bianchi *et al.* 2007)	RI lit [B] (V.I. Babushok *et al.* 2011)	RI lit other source [Ref]	Confirmed by Standard
75-07-0	Acetaldehyde	2.16	788				
123-38-6	Propionaldehyde	2.43	804	801			
123-72-8	Butyraldehyde	3.1	882	878			
66-25-1	Hexanaldehyde	6.76	1087	1080			
100-52-7	Benzaldehyde	15.05	1513	1528			
55012-32-3	Isopropyl benzaldehyde (cuminaldehyde)	18.91	1782		1784.1		
64-17-5	Ethanol	3.91	938	932			
78-92-2	2-Butanol	5.64	1027	1035			
71-23-8	1-Propanol	5.92	1043	1052			
78-83-1	2-Methyl-1-propanol	7.27	1112		1089.3*		
6032-29-7	2-Pentanol	7.83	1137	1142			
71-36-3	1-Butanol	8.29	1157	1152			
123-51-3	3-Methyl -1-butanol	9.51	1203	1215			
108-11-2	4-Methyl -2-pentanol	9.78	1218				
71-41-0	1-Pentanol	10.35	1248	1256			
626-89-1	4-Methyl -1-pentanol	11.55	1306				
543-49-7	2-Heptanol	11.59	1308		1315.3		
2313-61-3	4-Methyl -2-hexanol	11.66	1313				
111-27-3	1-Hexanol	12.24	1346	1354			
123-96-6	2-Octanol	13.4	1409				
3391-86-4	1-Octen-3-ol	13.9	1442	1456			yes

111-70-6	1-Heptanol	13.99	1448	1460			
104-76-7	2-Ethyl-1-hexanol	14.49	1479	1492			
111-87-5	1-Octanol	15.64	1552	1561			
15356-70-4	Cyclohexanol, 5methyl-2-(1-methylethyl)-,(1a,2b,5a)	16.9	1631		1630.4		
79-20-9	Ethanoic acid, methyl ester	2.69	846	828			
141-78-6	Ethanoic acid ethyl ester	3.21	890	893			
105-37-3	Propanoic acid ethyl ester	4.28	958				
97-62-1	2-Methylpropanoic acid ethyl ester	4.44	966	960			
109-60-4	Ethanoic acid propyl ester	4.6	974	976			
623-42-7	Butanoic acid methyl ester	4.79	982	982			
868-57-5	2-Methylbutanoic acid methyl ester	5.28	1003				
556-24-1	3-Methylbutanoic acid methyl ester	5.49	1017	1024			
105-54-4	Butanoic acid ethyl ester	5.87	1040	1040			
106-36-5	Propanoic acid propyl ester	6.04	1050	1056			
7452-79-1	2-Methylbutanoic acid ethyl ester	6.21	1060				
123-86-4	Ethanoic acid butyl ester	6.61	1080	1077			
624-24-8	Pentanoic acid methyl ester	6.87	1093	1087			
105-66-8	Butanoic acid propyl ester	7.64	1129	1123			
539-82-2	Pentanoic acid ethyl ester	7.92	1141	1138			
590-01-2	Propanoic acid butyl ester	8.06	1147				
97-87-0	Propanoic acid 2-methylbutyl ester	8.18	1152	1154			
539-90-2	Butanoic acid 2-methylpropyl ester	8.45	1163	1152			
628-63-7	Aethanoic acid pentyl ester	8.68	1172	1180			
123-92-2	Ethanoic acid 3-methylbutanyl ester	8.75	1175	1125			
106-70-7	Hexanoic acid methyl ester	9.01	1184	1190			
109-21-7	Butanoic acid butyl ester	9.68	1212	1223			
141-06-0	Pentanoic acid propyl ester	9.73	1215			1233 Peng et al.	
123-66-0	Hexanoic acid ethyl ester	9.99	1229	1238			
624-54-4	Propanoic acid pentyl ester	10.12	1236	1239			

109-19-3	3 -Methylbutanoic acid butyl ester	10.29	1244		
106-27-4	Butanoic acid 3-methylbutyl ester	10.6	1260	1267	
142-92-7	Ethanoic acid hexyl ester	10.73	1266	1269	
106-73-0	Heptanoic acid methyl ester	11.02	1280	1288	
591-68-4	Pentanoic acid butyl ester	11.5	1303		
5870-93-9	Butanoic acid heptyl ester	11.51	1304		
626-77-7	Hexanoic acid propyl ester	11.63	1311	1324	
106-30-9	Heptanoic acid ethyl ester	11.88	1325	1331	
4630-82-4	Cyclohexane carboxylic acid methyl ester	12.71	1371		
111-11-5	Octanoic acid methyl ester	12.82	1377	1387	
626-82-4	Hexanoic acid butyl ester	13.3	1403	1420	
108-64-5	3-Methylbutanoic acid ethyl ester	6.48	1074		
5870-93-9	Butanoic acid heptyl ester	11.51	1304		
3289-28-9	Cyclohexanecarboxylic acid ethyl ester	13.42	1411		
67-64-1	Acetone	2.61	814	814	yes
78-93-3	2-Butanone	3.37	901	901	
563-80-4	3-Methyl 2-butanone	4.56	972		
107-87-9	2-Pentanone	4.61	974	980	
431-03-8	2,3-Butanedione	4.61	974	986	
108-10-1	Methyl isobutyl ketone	5.19	998	1008	
591-78-6	2-Hexanone	5.21	999		
565-61-7	3-Methyl 2-pentanone	5.37	1009		
600-14-6	2,3- Pentanedione	6.33	1066	1071	
105-42-0	4-Methyl 2-hexanone	8.84	1178		
110-43-0	2-Heptanone	8.91	1180	1185	
110-93-0	6-Methyl -5-hepten-2-one	11.94	1329	1340	
107-92-6	Butanoic acid	16.82	1626	1630	yes
109-52-4	Pentanoic acid	18.4	1737		yes

142-62-1	Hexanoic acid	19.77	1854				
503-74-2	3-Methylbutanoic acid	17.33	1659				yes
64-19-7	Ethanoic acid	14.11	1455	1480			
65-85-0	Benzoic acid	27	2266				
79-09-4	Propanoic acid	15.42	1538	1554			
79-31-2	2-Methylpropanoic acid	15.93	1570				
111-14-8	Heptanoic acid	21.23	1972				
98-89-5	Cyclohexanecarboxylic acid	22.89	2081				
75-50-3	Trimethylamine	1.95	775				yes
124-40-3	Dimethylamine	3.19	889				
75-05-8	Acetonitrile	5.1	995				
926-64-7	Acetonitrile (dimethylamino)	10.02	1230			1243 (Pub Chem)	
7149-26-0	1,6-Octadien-3-ol, 3,7-dimethyl-2-aminobenzoate	15.63	1551				
120-72-9	Indole	26.78	2253				yes
83-34-1	3-Methylindole	27.27	2282				yes
75-18-3	Dimethyl sulphide	2.3	795				yes
624-92-0	Dimethyl disuphide	6.52	1076	1075			yes
5925-75-7	S-Methyl propanethioate	7.45	1121				
23747-45-7	S-Methyl 3-methylbutanethioate	9.69	1213				
2179-60-4	Methyl propyl disulphide	9.87	1223				
1618-26-4	2,4-Dithiapentane	10.6	1260			1260 (Shluter et al)	
57-06-7	Allyl isothiocyanate	12.32	1350	1372			
3658-80-8	Dimethyl trisulphide	12.65	1368	1383	1376.2		yes
75-09-2	Dichloromethane	3.78	930				
1073-91-2	1,2,4,5- Tetroxane, 3,3,6,6-tetramethyl	4.63	975				
7149-26-0	1,6-Octadien-3-ol, 3,7-dimethyl-2-aminobenzoate	15.63	1551				

140-67-0	Estragole	17.32	1658				
108-95-2	Phenol	21.84	1959				
106-44-5	4-Methylphenol	22.76	2014				
120-72-9	Indole	26.78	2253				yes
83-34-1	3-Methyl indole	27.27	2282				yes
	Terpene (1)	3.12	884				
	Terpene (2)	4.13	950				
	Terpene (3)	5.3	1005				
	Terpene (4)	5.52	1019				
	Terpene (5)	5.78	1035				
	Terpene (6)	6.39	1069				
	Terpene (7)	7.13	1105				
	Terpene (8)	7.37	1117				
	Terpene (9)	8.16	1151				
	Terpene (10)	8.42	1162				
	Terpene (11)	8.47	1164				
	Terpene (12)	9.16	1190				
	Terpene (13)	9.34	1196				
	Terpene (14)	9.92	1225				
	Terpene (15)	10.16	1238				
	Terpene (16)	10.38	1249				
	Terpene (17)	10.63	1261				
	Terpene (18)	10.95	1277				
	Terpene (19)	13.69	1428				
	Terpene (20)	13.73	1431				
	Terpene (21)	14.56	1483				
	Terpene (22)	14.73	1493				
	Terpene (23)	15.47	1541				
	Terpene (24)	16.16	1584				

	Terpene (25)	16.35	1595
	Terpene (26)	17.99	1700
	Terpene (27)	18.12	1712
	Terpene (28)	18.22	1721
	Terpene (29)	20.71	1929
	Terpene (29)	23.86	2079
	Terpene (30)	24.64	2125
	Unidentified (1)	3.46	908
	Unidentified (2)	3.81	931
	Unidentified (3)	4.38	963
	Unidentified (4)	5.19	998
	Unidentified (5)	5.37	1009
	Unidentified (6)	5.84	1039
	Unidentified (7)	5.87	1040
	Unidentified (8)	6.04	1050
	Unidentified (9)	6.23	1061
	Unidentified (10)	6.36	1068
	Unidentified (11)	6.67	1083
	Unidentified (12)	6.92	1095
	Unidentified (13)	7.6	1127
	Unidentified (14)	7.91	1141
	Unidentified (15)	9.58	1207
	Unidentified (16)	10.43	1252
	Unidentified (17)	10.53	1257
	Unidentified (18)	11.26	1291
	Unidentified (19)	12.04	1334
	Unidentified (20)	12.29	1349
	Unidentified (21)	13.31	1403
	Unidentified (22)	13.35	1406

	Unidentified (21)	15.02	1512
	Unidentified (22)	17.58	1675
	Unidentified (23)	17.75	1685
	Unidentified (24)	19.86	1861
	Unidentified (25)	20.31	1896
	Unidentified (26)	20.82	1938
	Unidentified (27)	21.22	1971
	Unidentified (28)	21.56	1998
	Unidentified (29)	21.99	2010
	Unidentified (30)	25.61	2183
	Unidentified (31)	25.88	2199
	Unidentified (32)	26.97	2264

Table S13 shows the retention indices calculated from the experimental data and where possible compared to available literature retention indices for PEG based polar columns.

[A] F. Bianchi *et al.* J. Sep. Sci. 2007, 30, 563-572 **[B]** V.I. Babushok *et al.* J. Phys. Chem. Ref. Data, Vol. 40, No. 4, 2011, 043101-1, C.T.Peng *et al.*, J. Chromatogr., 1991, 586, 1, 85-112, S. Schlüter *et al.*, J. Agric. Food Chem., 1999, 47, 12, 5146-5150

Sample	Diet	Origin	Dimethyl Sulphide	Acetone	Butanoic acid, ethyl ester	2-Methyl-butanoic acid, ethyl	Dimethyl disulphide	1-Octen-3-one	Dimethyl Trisulphide	1-Octen-3-ol	Ethanoic acid	Butanoic acid	3-Methylbutanoic acid	Pentanoic acid	Indole	3-methyl indole	Trimethyl amine
1	Veg	UK	5238.15	598.82	0	0	148.61	11.88	43.95	35.07	4297.67	2492.6	63.56	88.3	2213.48	626.3	180.94
2	Meat	UK	916.89	1478.2	40.47	0	285.53	0	89.11	58.2	4989.53	2681.27	642.62	816.26	2342.28	830.68	132.49
3	Veg	UK	1328.85	1844.24	175.28	13.47	222.07	0	76.82	26.05	6193.06	2785.31	826.22	1113.08	2862.26	726.45	304.47
4	Veg	UK	153.99	1098.05	39.28	0.34	118.12	0	52.36	38.05	9030	4103.38	1074.09	1760.06	1508.71	670.22	
5	Veg	UK	502.9	1539.91	39.33	0.84	233.34	0	68.58	12.27	6953.33	4177.96	422.29	734.63	1862.04	1040.24	163.22
6	Meat	UK	669.77	2222.98	311.08	16.6	245.71	0	53.77	3.03	672	3328.19	991.47	1010.12	2500.71	585.03	
7	Meat	UK	8088.71	2198.58	339.64	18.1	295.84	0	119.51	2.37	6087.5	5548.11	2295.07	2380.76	2910.77	425.42	920.5
8	Meat	UK	5408.83	869.02	264.7	8.14	75.94	0	30.22	2.7	9146.98	7427.16	3602.64	5970.98	3318.1	677.29	
9	Veg	UK	8636.56	1356.9	0	0	91.31	0	19.23	0	11343.44	8228.25	406.55	558.75	4309.01	1251.73	
10	Veg	UK	1199.38	1494.35	43.41	4.66	48.47	0	10.83	0	8830	7700.68	372.04	565.21	3278.59	1629.85	
11	Meat	UK	4368.62	697.32	129.1	8.29	863.74	0	253.34	1.08	7236.49	6463.85	1927.86	2984.45	3247.19	3054	
12	Veg	UK	91.42	442.04	1659.32	0	52.95	0	22.66	0	8685.19	7331.51	2224.45	3531.9	2698.7	2185.55	
13	Veg	UK	565.56	874.6	1166.05	0	590.75	0	183.08	1.83	7007.78	7496.09	2506.21	4943.48	3577.83	1529.18	

14	Meat	UK	341.06	2520.59	69.78	15.41	725.96	0	135.15	0	10337.8	8376.46	366.05	730.37	4127.2	1457.4	
15	Meat	UK	25.8	1049.61	53.49	0	62.03	0	10.15	0	9133.87	6881.94	1707.53	2708.97	3961.73	2583.96	
16	Meat	UK	2055.58	1086.78	2468.23	179.98	755.98	0	141.86	0	8462.22	7518.38	2386.87	3360.7	3320.22	2180.09	
17	Meat	South America	746.73	2376.78	0	0	315.09	0	62.65	0.42	9434.78	9009.96	3084.57	4667.99	4313.84	1485.49	175.92
18	Meat	South America	6961.49	819.28	828.15	42.43	1012.95	0	315.66	0	9154.69	9363.66	0	6012.64	4756.23	1472.41	185.18
19	Meat	South America	1333.53	1011.08	0	0	957.42	0	207.74	1.23	7967.65	8624.19	5645.87	21886	4443.32	2561.2	
20	Meat	South America	1910.67	2048.95	0	0	373.75	0	199.6	0	8137.5	8632.79	575.85	946.12	3836.98	1085.39	
21	Meat	South America	621.54	1369.21	0	0	145.66	0	30.67	0	10750	8153.84	220.33	3015.06	3286.3	1646.75	
22	Meat	South America	5311.77	1440.22	0	0	355.64	0	91.73	0	9250	8547.05	5508.16	9259.46	3465.66	1162.92	5311.77
23	Meat	South America	0	1993.51	0	0	36.04	0	12.65	0	10519.44	8157.61	716.06	1117.26	4232.06	3483.23	351.83
24	Meat	South America	1376.92	1768.06	0	0	1302.92	0	241.31	0	9611.54	9050.63	6885.2	11172.57	3701.86	2103.59	104.16
25	Meat	South America	1294.29	1347.73	392.53	8.98	121.09	0	50.73	0	12963.32	7042.55	4838.42	6711.54	4677.54	1622.99	158.33
26	Veg	South America	8078.67	2000.63	0	0	712.09	0	142.55	2.22	8686.36	9953.48	5095.05	10216.02	5477.47	1479.56	39.88
27	Veg	Africa	611.89	1370.4	0	0	619.84	0	162.57	0	9590	9784.61	1836.05	2015.14	4776.4	1389.45	
28	Meat	Asia	613.44	453.09	1770.12	0	98.52	0	36.1	0	9975	8838.37	0	0	5050.3	956.18	
29	Veg	Africa	2704.33	2287.17	0	0	227.25	0	33.37	0	9203.51	9344.76	5220.03	6311.4	4543.73	2267.31	
30	Meat	Europe	1532.28	785.92	0	0	963.02	0	319.58	0	7670.83	4368.24	8262.24	13774.4	5070.29	790.31	
31	Meat	Africa	579.1	2156.48	0	0	1137.74	0	409.57	0	9670.6	7391.69	4914.36	4364.38	4714.12	1085.59	
32	Meat	Africa	5747.89	1747.46	2333.28	0	828.49	0	286.25	0	9475.73	9324.14	3606.73	3163.39	4117.27	774.76	
33	Veg	Asia	124.57	957.07	290.43	0	37.16	0	15.66	0	9763.67	8923.94	3558.52	3050.35	4586.6	686.74	
34	Meat	Asia	1546.13	1845.72	859.88	0	35.01	0	14.89	0	9982.29	8872.95	4098.55	5810.87	4605	403.99	545.95
35	Meat	Asia	2070.65	3005.62	0	0	1189.27	0	362.32	0	9984.12	9632.85	4149.28	5829.42	4782.7	993.61	
36	Meat	South America	4157.13	1998.93	118.41	0	1084.08	0	364.48	0	8937.71	9604.83	4732.02	7429.37	3725.53	636.13	
37	Veg	Europe	25626.1	1101.97	0	0	1058.28	0	199.02	0	12233.44	10448.77	3041.42	3295.83	289.94	37.25	55.35
38	Veg	Europe	3689.88	1028.97	0	0	809.81	0	162.32	0	11374.26	11552.97	5821.2	5532.56	4770.44	717.13	119.94

Table S14 Raw data of concentrations of a range of stool compounds, in ng/g, calculated by adding ^{13}C labelled internal standards to stool samples and analysing the headspace using automated thermal desorption gas chromatography mass spectrometry.

CAS	Nitrogen containing compounds	RT (mins)	1	2	5	7	17	18	22	23	24	25	26	32	34	37	38
75-50-3	Trimethylamine	1.95	8.26	7.76	31.95	11.95	7.42	8.39	4.19	18.21	40.65	40.01	97.26	5.65			1.59
124-40-3	Dimethylamine	3.19						9.51	215.77			314.32			86.69		
75-05-8	Acetonitrile	5.1	16.55	10.99		15.94	17.05	13.13		21.01	13.99	8.53	12.68	8.51	5.47	16.82	21.23
926-64-7	Acetonitrile (dimethylamino)	10.02	6.41	51.86			7.94	22.61	32.54	4.41	23.03			13.57	8.64	21.88	13.52
120-72-9	Indole	26.78	58.63		31.28		62.31	39.9	23.14	11.88	39.84	30.13	46.36	15.01	56.04		
83-34-1	3-Methylindole	27.27						26.31		30.13	3.66			23.48			

Tables S15 Nitrogen containing compounds found in stool samples adjusted to pH 13 with sodium hydroxide for 15 participants with retention time and chromatographic peak area.

CAS	Aldehydes	RT (mins)	1	2	5	7	17	18	22	23	24	25	26	32	34	37	38
75-07-0	Acetaldehyde	2.16	12.37	2.08	4.31	31.59	2.23	10.56	1.84	2.45		19.32	9.04	5.36	36.89	5.16	5.1
123-72-8	Butyraldehyde	3.1	25.28				17	1.85					8.34		3.65	1.89	
66-25-1	Hexanaldehyde	6.76	5.29														

Tables S16 Aldehyde compounds found in stool samples adjusted to pH 13 with sodium hydroxide for 15 participants with retention time and chromatographic peak area.

CAS	Sulphides	RT (mins)	1	2	5	7	17	18	22	23	24	25	26	32	34	37	38
75-18-3	Dimethyl sulphide	2.3	110.78	32.67	17.83	104.6	4.69	243.23	102.33		16.1	73.12	115.65	3.39	34.36	332.31	12.93
624-92-0	Dimethyl disuphide	6.52	41.24				50.8	148.71	21.35	4.85	51.92	83.43	46.39	14.18			46.16
23747-45-7	S-methyl 3-methylbutanethioate	9.69					3.1										

Tables S17 Sulphide compounds found in stool samples adjusted to pH 13 with sodium hydroxide for 15 participants with retention time and chromatographic peak area.

CAS	Ketones	RT (mins)	1	2	5	7	17	18	22	23	24	25	26	32	34	37	38
67-64-1	Acetone	2.61	80.3	200.02	179.45	174.92	434.16	69.08	46.68	314.15	117.53	234.79	82.31	59.39	105.27	196.82	55.35
107-87-9	2-pentanone	4.61		27.89	48.5		133.37			32.29			13.75			19.26	
591-78-6	2-hexanone	6.75					28.63			14.89							
105-42-0	4-Methyl 2-hexanone	8.84				3.73	14.3										
110-43-0	2-Heptanone	8.91								3.19							
111-13-7	2-Octanone	10.91			14.42												
110-93-0	6-Methyl 5-hepten-2-one	11.94	58.17	64.95	9.81	2.93	9.01	7.72	3.28	17.36	67.41	32.06	9.16	10.43	3.52	3.78	52.32

Tables S18 Ketone compounds found in stool samples adjusted to pH 13 with sodium hydroxide for 15 participants with retention time and chromatographic peak area.

CAS	Esters	RT (mins)	1	2	5	7	17	18	22	23	24	25	26	32	34	37	38
79-20-9	Ethanoic acid, methyl ester	2.69	67.5	117.34	74.36	72.72	84.96	56.36	70.26	52.96	58.36		78.99	85.11	394.92	48.61	61.7
141-78-6	Ethanoic acid ethyl ester	3.21				40.51	99.29							12.32			
105-37-3	Propanoic acid ethyl ester	4.28						39.5				448.29		26.95	886.65		
97-62-1	Propanoic acid, 2-methyl- ethyl ester	4.44						27.03				53.51			39.29		
109-60-4	Ethanoic acid propyl ester	4.6				62.1						200.57			319.61		
623-42-7	Butanoic acid methyl ester	4.79				223.7		87.46	17.03	5.14	5.03	778.65	2.21	115.95	1265.08		
868-57-5	2-Methylbutanoic acid methyl ester	5.28				20.92		24.95				19.76		16.28	4.18		
556-24-1	3-Methylbutanoic acid methyl ester	5.49				27.8											
105-54-4	Butanoic acid methyl ester	5.87				42.67		208.41	51.57		55.49	2199.01		78.62			
106-36-5	Propanoic acid propyl ester	6.04										23.62		8.07	268.18		
7452-79-1	2-Methylbutanoic acid ethyl ester	6.21				26.14		25.14	6.42			62.44		7.04	25.28		
108-64-5	3-Methylbutanoic acid ethyl ester	6.48		13.13	30.12	110									51.64	50.48	
123-86-4	Ethanoic acid butyl ester	6.61										127.97			182.48		
624-24-8	Pentanoic acid methyl ester	6.87				32.36		25.41						14.08	135.44		
105-66-8	Butanoic acid propyl ester	7.64				89.12		45.96	7.95		5.63	560.89		9.22	578.32		
539-82-2	Pentanoic acid ethyl ester	7.92										52.24		5.65	593.2		
590-01-2	Propanoic acid butyl ester	8.06													134.41		
97-87-0	Propanoic acid 2-methyl butyl ester	8.18													2.28		
539-90-2	Butanoic acid 2-methylpropyl ester	8.45						11.43	1.78								
628-63-7	Ethanoic acid pentyl ester	8.68													13.76		
123-92-2	Ethanoic acid 3-methyl butanyl ester	8.75															
106-70-7	Hexanoic acid methyl ester	9.01				6.03											
109-21-7	Butanoic acid butyl ester	9.68				26.75		26.14	5.78			306.99		3.26	178.47		
141-06-0	Pentanoic acid propyl ester	9.73									5.67						1.28
123-66-0	Hexanoic acid ethyl ester	9.99				11.1							49.67				
109-19-3	3- Methylbutanoic acid butyl ester	10.29			10.69							33.21				4.74	
591-68-4	Pentanoic acid butyl ester	11.5						2.51				11.79					
5870-93-9	Butanoic acid heptyl ester	11.51													15.71		
4630-82-4	Cyclohexanecarboxylic acid methyl ester	12.65										33.8					
3289-28-9	Cyclohexanecarboxylic acid ethyl ester	13.42										44.71					

Tables S19 Ester compounds found in stool samples adjusted to pH 13 with sodium hydroxide for 15 participants with retention time and chromatographic peak area.

CAS	Alcohols	RT (mins)	1	2	5	7	17	18	22	23	24	25	26	32	34	37	38
64-17-5	Ethanol	3.91				128.5		161.7		122.42		74.94	222.59	217.58	1184.95		
78-92-2	2-Butanol	5.64					13.61										
73-23-8	1-Propanol	5.92													3777.17		206.89
78-83-1	2-Methyl -1-propanol	7.27						19.44	14.18		20.11			7.69	15.82		
1572-93-6	3-Methyl-2-butanol	7.78									2.06			1.81			
6032-29-7	2-Pentanol	7.83				3.93											
71-36-3	1-Butanol	8.29						29.81	24.73		32.63	320.62	6.55	19.73	438.54	5.74	10.95
137-32-6	2-Methyl-1-butanol	9.51	5.18			14.19	13.4	11.57	9.12	7.94	22.76	12.4	9.53	5.48		10.37	9.62
71-41-0	1-Pentanol	10.35				13.3	9.36	10.46	8.03		14.41			6.98			
626-89-1	4-Methyl-1-pentanol	11.51									1.49						

Tables S20 Alcohols found in stool samples adjusted to pH 13 with sodium hydroxide for 15 participants with retention time and chromatographic peak area.

CAS	Acids	RT (mins)	1	2	5	7	17	18	22	23	24	25	26	32	34	37	38
79-31-2	2-Methylpropanoic acid	6.7						4.68									
64-19-7	Ethanoic acid	14.11	26.14		19.23	31.94	15.4	19	18.72		12.84	16.95		20.32	36.52	20.27	23.15
107-92-6	Butanoic acid	16.82	18.14	13.15		24.67									34.47	10.52	

Tables S21 Acids found in stool samples adjusted to pH 13 with sodium hydroxide for 15 participants with retention time and chromatographic peak area.

CAS	Aromatic compounds	RT (mins)	1	2	5	7	17	18	22	23	24	25	26	32	34	37	38
7149-26-0	1,6-Octadien-3-ol, 3,7-dimethyl-,2-aminobenzoate	15.63													52.57		
108-95-2	Phenol	21.84	6.32	7.81	6.18	5.94	8.59	7.38	19.75	5.15	9.6	9.98	10.15	5.66	6.85	5.38	4.77
106-44-5	4-Methylphenol	22.76	59.39	70.1	100.66	60.66	120.73	69.62	76.09	83.48	113.1	35.04	34.77	49.98	30.72	40.6	44.75
120-72-9	Indole	26.78	58.63		31.28		62.31	39.9	23.14	11.88	39.84	30.13	46.36	15.01	56.04		
83-34-1	3-Methylindole	27.27						26.31		30.13	3.66			23.48			

Tables S22 Aromatic compounds found in stool samples adjusted to pH 13 with sodium hydroxide for 15 participants with retention time and chromatographic peak area.

Siloxanes	RT (mins)	1	2	5	7	17	18	22	23	24	25	26	32	34	37	38
Siloxane (1)	4.31	23.43	33.97	17.49	110.4	24.75			15.45	31.05		11.31			12.6	
Siloxane (2)	6.83					5.38					16.75					
Siloxane (3)	7.58			3.72		1.97									2.78	
Siloxane (4)	10.53								5.21					13.13		
Siloxane (5)	13.03											5.63				
Siloxane (6)	13.13	9.95	7.58	3.97	3	5.61	2.94	2.32	2.98	10.19	2.29		5.07		3.18	2.34
Siloxane (7)	15.32	8.53												9.16		

Tables S23 Siloxane compounds found in stool samples adjusted to pH 13 with sodium hydroxide for 15 participants with retention time and chromatographic peak area.

Terpenes	RT (mins)	1	2	5	7	17	18	22	23	24	25	26	32	34	37	38
Terpene (1)	3.02									29.63			6.39			
Terpene (2)	5.3			5.08			46.18			1.12						
Terpene (3)	5.52	78.67	332.62	68.07		46.87			67.66	39.98	68.77	28.22	31.64	49.67	13.5	
Terpene (4)	5.59	22.2						21.66			16.54		11.15			
Terpene (5)	5.78	10.31	64.32	20.43		46.26			16.12			32.03			26.52	
Terpene (6)	6.39		10.4	19.85					8.51			10.2		29.68		
Terpene (7)	6.87	6.57														
Terpene (8)	7.13	61.47	61.46	145.04	10.19	56.33	7.64		22.71		112.28	17.89	24.61	12.1	10.33	
Terpene (9)	7.37	45.91	32.57	11.38	8.62	1.23	6.1		8.66		32.81			15.76		18.15
Terpene (10)	7.4								11.42				7.58			
Terpene (11)	7.86					155.85	55.22	3.02	1.26	3.18		1.78				
Terpene (12)	7.99	72.26	72.86	24.83				2.18	34.55	5.4	179.23	4.52	15.04		2.89	
Terpene (13)	8.16		68.87	67.35		7.81	1.56	14.58	15.06	1.21	17.75	14.04			30.04	12.98
Terpene (14)	8.37								3.6							
Terpene (15)	8.42	2.1	698.76			39.18							5.84	510.92	3.02	
Terpene (16)	8.47											1.27				
Terpene (17)	8.69	3.27	6.79						4.23		83.44					
Terpene (18)	8.88			21.35							3.81	3.07	1.29		3.78	
Terpene (19)	9.16	74.57		64.69	21.49	64.24	10.9	3.57	28.66	31.28	470.09	5.61	21.22	1328.47	39.74	57.75
Terpene (20)	9.34	7.38	1486.21	7.54		2.88	1.12		5.5	2.77	5.39	3.76	1.95	50.39		
Terpene (21)	9.92													47.96		
Terpene (22)	10.16	11	18.56	99.67				0.26	7.45		105.84		28.74	136.34		5.77

Terpene (23)	10.38	5.71							3.8			4.3				
Terpene (24)	10.51	14.65		71	11.78	14.21	12.55	10.09		10.03	45.09	8.68			4.64	7.23
Terpene (25)	10.6								11.55				28.98			
Terpene (26)	10.63		70.69													
Terpene (27)	10.83		25.54													
Terpene (28)	10.95		17.05			4.81										
Terpene (29)	12.21			9.89	4.84	3.82	3.69				3.08	3.42			1.4	4.8
Terpene (30)	12.53		4.4		4.88									24.63		
Terpene (31)	12.92		10.4													
Terpene (32)	13.69		24.13	1.13												
Terpene (33)	14.73			2.13											5.32	
Terpene (34)	15.47				7.93	14.08	5.27	6.54				12.65			6.84	5.59
Terpene (35)	16.35	14.94	23.15	18.19		5.74	4.67		8.71		10.84	4.41	4.21		18.77	
Terpene (36)	17.46		4.93	3.72		3.89	3.85				3.67	3.56	4.61		4.19	5.81
Terpene (37)	17.99			1.67												
Terpene (38)	18.12			2.42												
Terpene (39)	18.22			3.12												
Terpene (40)	19.58		12.23													
Terpene (41)	20.71	40.4	39.44	22.93	39.12	45.63	29.39	26.86	23.71	22.01	25.07	5.19	17.4	39.5	33.56	26.66
Terpene (42)	23.86		3.14	1.88	1.05		1.46	1.5		3.03	1.61				2.8	
Terpene (43)	24.64		0.75	0.64	0.58		0.69	0.55	0.72	0.92	0.65		0.74		1.13	

Tables S24 Terpene compounds found in stool samples adjusted to pH 13 with sodium hydroxide for 15 participants with retention time and chromatographic peak area.

Unidentified	RT (mins)	1	2	5	7	17	18	22	23	24	25	26	32	34	37	38
Unidentified (1)	4.56	8.29					10.18	9.11		24.2			19.73			
Unidentified (2)	6.08								0.82							
Unidentified (3)	6.23															2.29
Unidentified (4)	6.67			13.68						28.6						
Unidentified (5)	7.55	2.5														
Unidentified (6)	7.6											2.46				
Unidentified (7)	8.39									2.3						
Unidentified (8)	9.58			14.62										13.56		
Unidentified (9)	11.53				2.02											

Unidentified (10)	12.29														3.63	
Unidentified (11)	14.25											13.69				
Unidentified (12)	15.49												4.91			
Unidentified (13)	16.84			9.66		11.58	12.11	13.6	8.33	12.44	13.74	8.37	12.35			13.09
Unidentified (14)	17.38				7.93				2.84					9.72		
Unidentified (15)	18.38		3.88		9.29	2.78	8.64	8.27			7.29	2.1			2.14	6.53
Unidentified (16)	19.86							0.8								

Tables S25 Unidentified compounds found in stool samples adjusted to pH 13 with sodium hydroxide for 15 participants with retention time and chromatographic peak area.

a)

b)

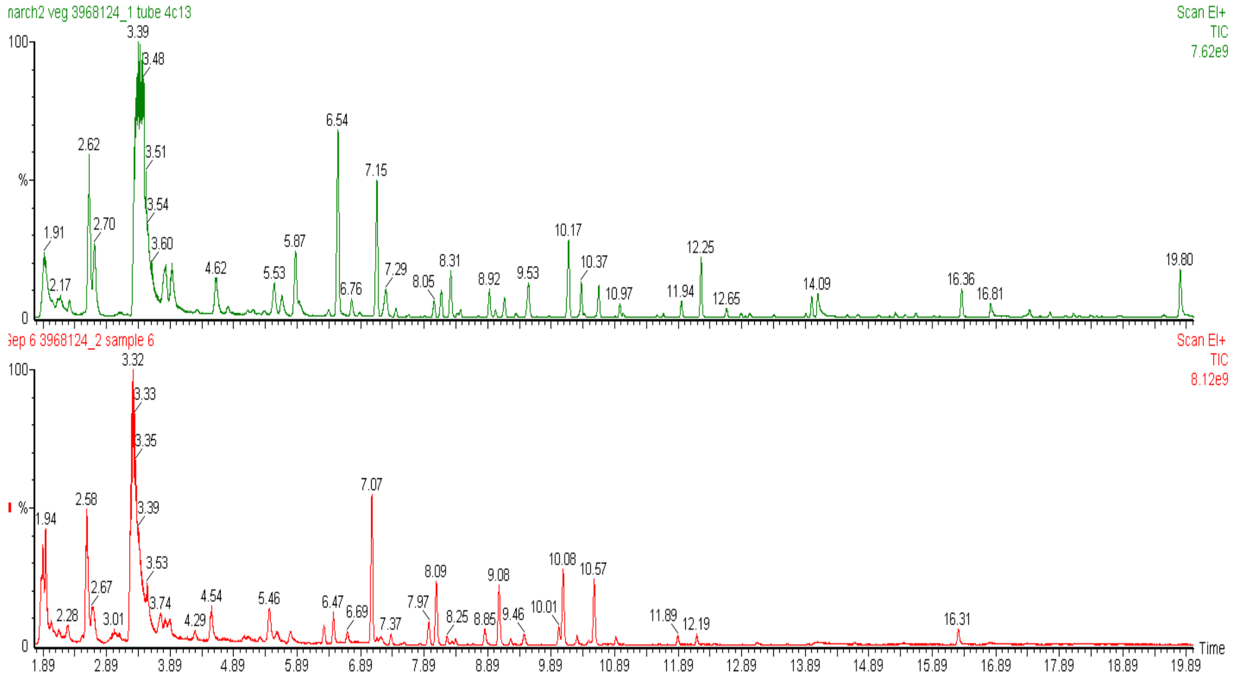

Figure S1 a) Chromatogram produced from unmodified stool sample spiked with ^{13}C labelled internal standards (solution 1) b) chromatogram produced from another aliquot of the sample with 5mL of 0.1M aqueous sodium hydroxide added in addition to ^{13}C labelled trimethylamine.

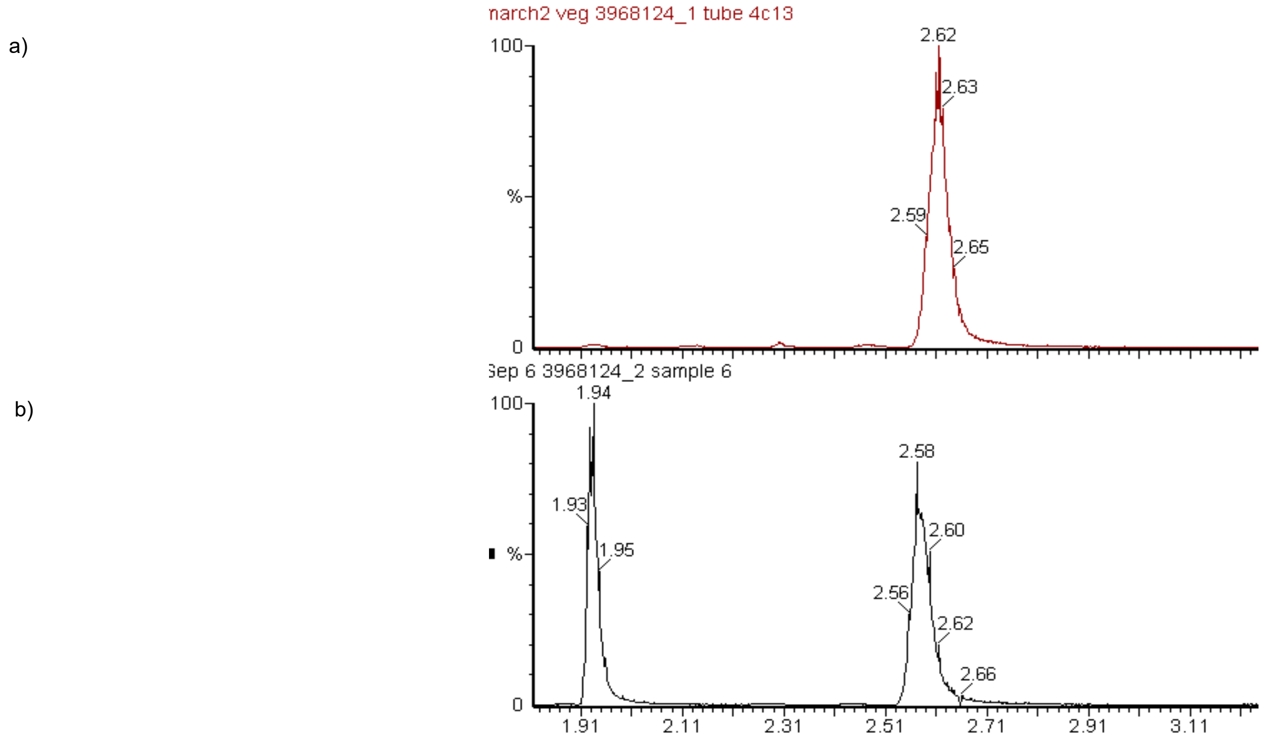

Figure S2 a) the same sample as figure S1a with only m/z 58 ions displayed vs. time. The peak at retention time 2.62 min was identified as acetone b) the same sample as Figure S1b (the pH altered stool) with only m/z 58 ions displayed vs. time. The peak at retention time 1.94 min was identified by the NIST library as trimethylamine, and the peak at retention time 2.58 min as acetone.

Compound	2	6	7	8	11	14	15	16	Mean omnivore	1	3	4	5	9	10	12	13	Mean vegetarian
Trimethylamine	ND	ND	ND	ND	ND	2.6	ND	ND	**2.6**	ND	ND	ND	ND	ND	ND	ND	ND	**ND**
Dimethyl Sulphide	213.6	400.0	1786.2	850.4	3922.0	50.5	34.9	827.1	**1010.6**	2159.0	229.1	28.0	178.6	2423.1	260.2	314.5	621.3	**776.7**
Acetone	290.4	2384.9	3771.6	1666.2	602.6	2149.0	72.6	145.2	**1385.3**	842.2	2029.2	410.2	1847.7	838.5	254.1	653.4	700.6	**947.0**
Propanethiol	9.5	ND	ND	23.8	ND	33.3	ND	ND	**22.2**	ND	ND	ND	ND	7.6	ND	ND	ND	**7.6**
Pantanal	ND	ND	ND	ND	ND	ND	ND	ND	**ND**	ND	ND	ND	ND	ND	ND	ND	ND	**ND**
Ethyl-2-methyl butanoate	ND	ND	ND	ND	ND	ND	ND	ND	**ND**	ND	ND	ND	ND	ND	ND	ND	ND	**ND**
Dimethyl disulphide	365.0	206.0	223.7	164.8	1106.7	129.5	64.8	688.8	**368.7**	288.5	482.7	14.1	223.7	88.3	306.1	100.1	370.9	**234.3**
1-Octen-3-one	ND	ND	ND	ND	ND	ND	ND	ND	**ND**	ND	2.4	1.3	ND	ND	ND	ND	2.8	**2.1**
Dimethyl trisulphide	94.7	78.9	98.6	68.7	560.3	51.3	5.5	284.1	**155.3**	75.0	161.8	5.5	56.0	20.5	75.0	39.5	94.7	**66.0**
1-Octen-3-ol	ND	ND	ND	ND	0.2	ND	ND	ND	**0.2**	0.2	ND	ND	ND	ND	ND	ND	0.2	**0.2**

Butanoic acid	88.1	363.5	82.6	170.7	269.8	19.8	66.1	253.3	**164.2**	27.5	82.6	44.1	38.5	25.9	38.5	1921.9	126.7	**288.2**
3-Methylbutanoic acid	23.0	140.4	25.5	70.2	44.7	3.8	22.3	51.1	**47.6**	5.7	23.6	10.9	6.4	7.0	6.4	197.9	25.5	**35.4**
Pentanoic acid	12.8	98.9	19.1	59.4	36.4	ND	9.6	44.7	**40.1**	3.2	14.7	6.4	6.4	3.8	4.5	217.0	17.9	**34.2**
Indole	6.6	22.9	14.8	46.5	9.6	13.3	7.4	11.1	**16.5**	31.0	14.0	1.5	22.1	11.1	5.2	25.8	11.8	**15.3**
3-Methyl indole	9.8	11.5	5.7	29.5	57.4	4.9	32.0	13.1	**20.5**	11.5	12.3	16.4	24.6	19.7	18.0	8.2	11.5	**15.3**

Table S26 quantities of compounds determined from the headspace of healthy stool samples from the U.K. omnivore and vegetarian samples. Quantities were calculated by collecting a known volume of headspace on TD tubes and using calibration curves of standards spiked onto the same TD tubes at different concentrations to calculate concentration in ng/g of stool. N.D. =not detected.

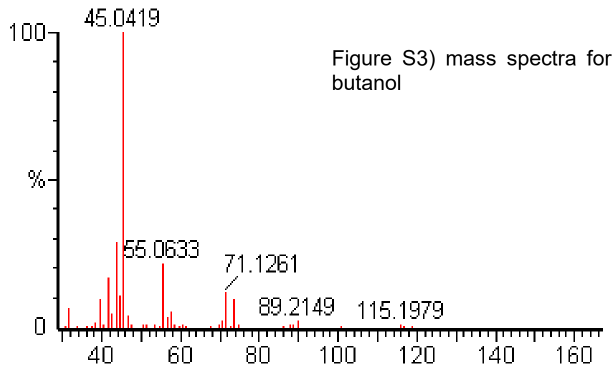

Figure S3) mass spectra for 3-methyl 2-butanol

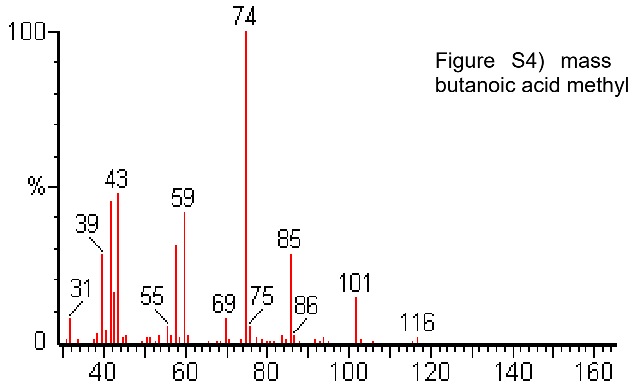

Figure S4) mass spectra for 3-methyl butanoic acid methyl ester

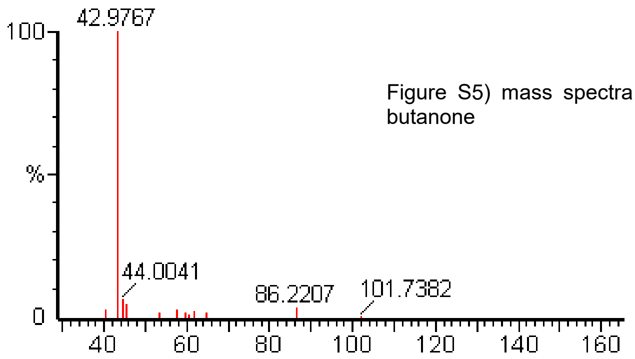

Figure S5) mass spectra for 3-methyl-2-butanone

Figure S6) mass spectra for propanoic acid 2-methylbutyl ester

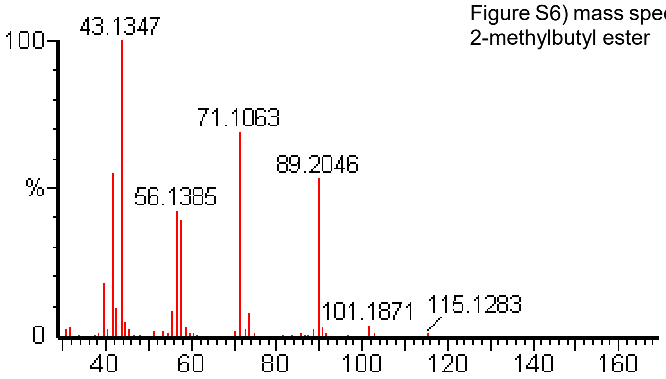

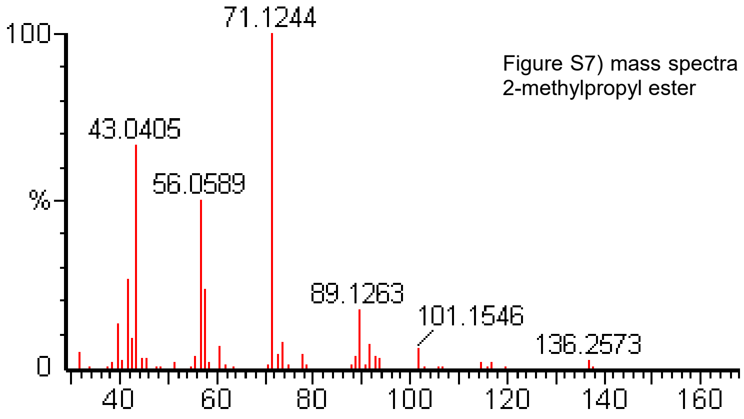

Figure S7) mass spectra for butanoic acid 2-methylpropyl ester

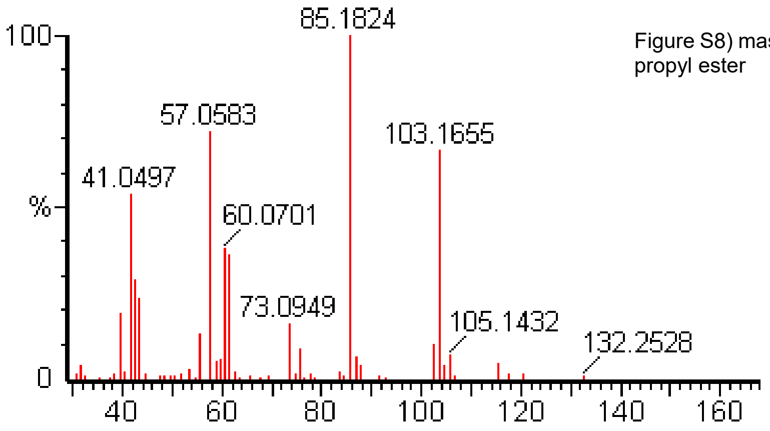

Figure S8) mass spectra for pentanoic acid propyl ester

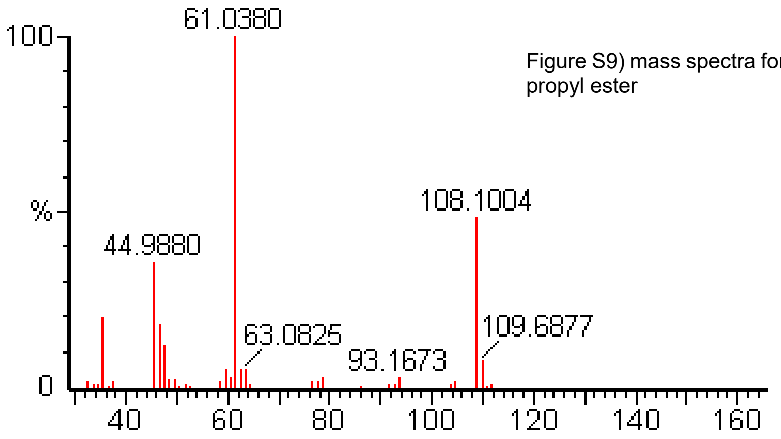

Figure S9) mass spectra for pentanoic acid propyl ester

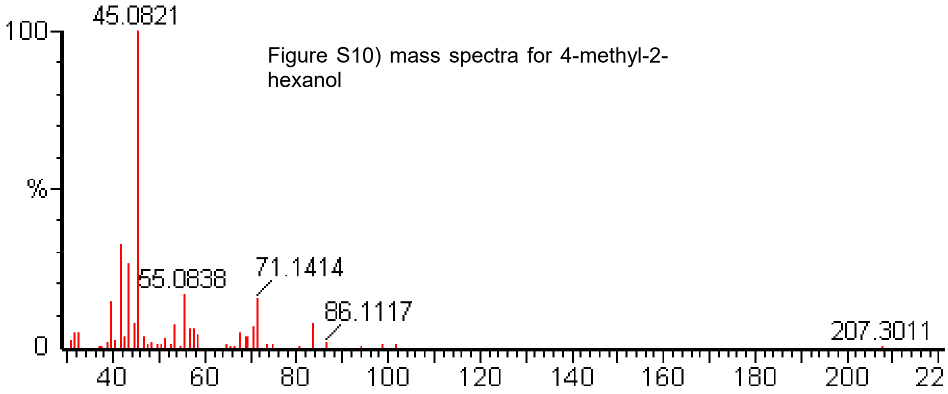

Figure S10) mass spectra for 4-methyl-2-hexanol

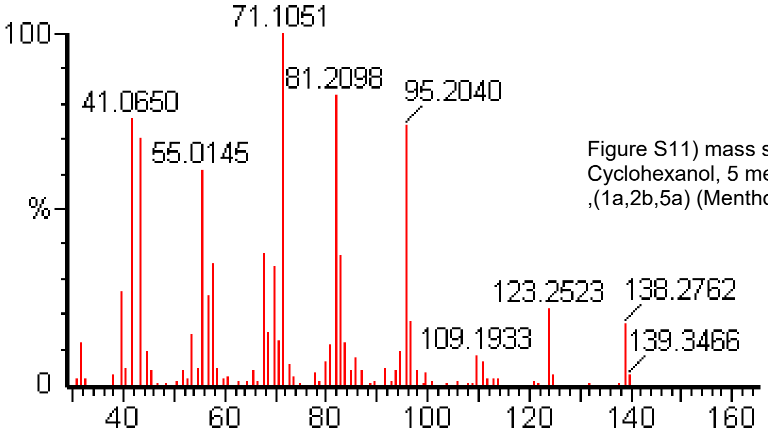

Figure S11) mass spectra for Cyclohexanol, 5 methyl-2-(1-methylethyl)-,(1a,2b,5a) (Menthol)

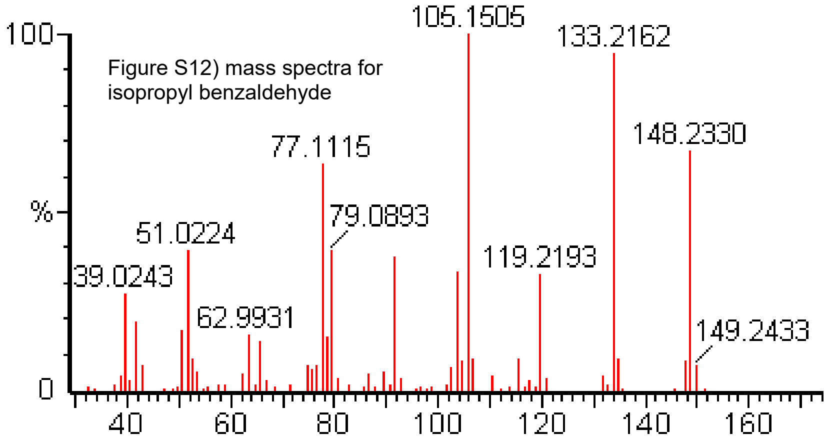

Figure S12) mass spectra for isopropyl benzaldehyde

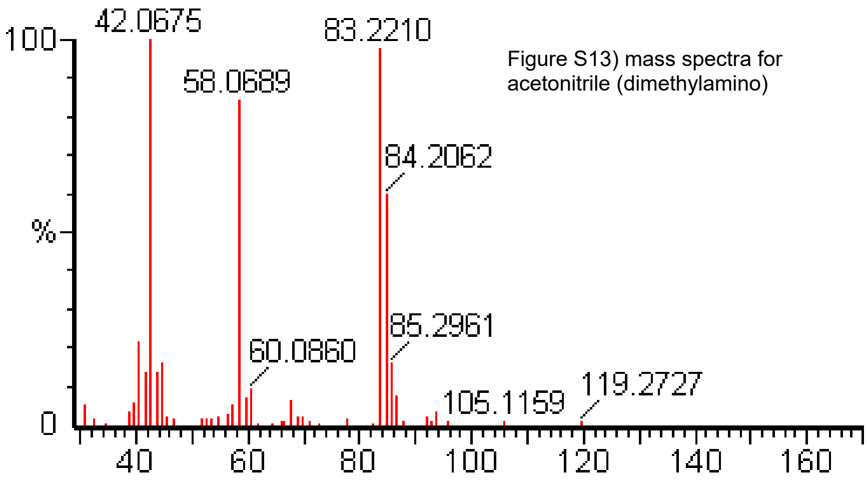

Figure S13) mass spectra for acetonitrile (dimethylamino)

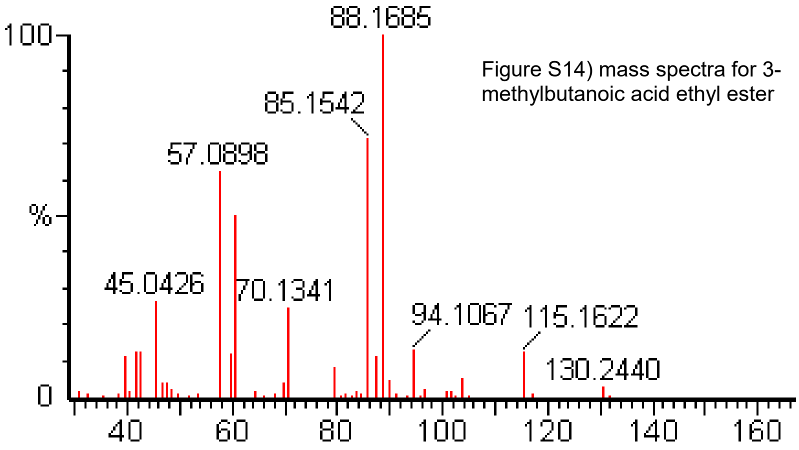

Figure S14) mass spectra for 3-methylbutanoic acid ethyl ester

Compound ^{13}C (purity)	Supplier	Volume/ mass (in 50mL methanol)
**Acetone 2-^{13}C (99%)**	Sigma Aldrich Company	50µL/39.55mg
**Ethyl butanoate 1-^{13}C (>95%)**	Precursor supplier, Cambridge Isotope Laboratories Ltd	50µL/43.25mg
**Dimethyl disulphide $^{13}C2$ (99%)**	Cambridge Isotope Laboratories Ltd	50µL/53.0mg
**Ethanoic acid 1-^{13}C (99%)**	Cambridge Isotope Laboratories Ltd	50µL/52.5mg
**Butanoic acid 1-^{13}C (99%)**	Cambridge Isotope Laboratories Ltd	50µL/47.5mg
**3-Methylbutanoic acid 1-^{13}C (99%)**	Cambridge Isotope Laboratories Ltd	50µL/46.25mg
**Indole 2-^{13}C (98%)**	Cambridge Isotope Laboratories Ltd	80mg (solid)

Table S27 Solution 1: ^{13}C labelled compounds used as internal standards, purity, suppliers, and masses used.

CAS number	Compound	Supplier	Volume/ mass (in 100mL methanol*)
**75-18-3**	Dimethyl sulphide	Sigma Aldrich Company	100µL/84.6mg
**67-64-1**	Acetone	Sigma Aldrich Company	100µL/79.1mg
**7452-79-1**	2-Methyl-butanoic acid, ethyl ester	Sigma Aldrich Company	100µL/86.5mg
**624-92-0**	Dimethyl disulphide	Sigma Aldrich Company	100µL/106.0mg
**4312-99-6**	1-Octen-3-one	Sigma Aldrich Company	100µL/84.3mg
**3658-80-8**	Dimethyl trisulphide	Sigma Aldrich Company	10µL/15.97mg (in75mL methanol)
**3391-86-4**	1-Octen-3-ol	Sigma Aldrich Company	100µL/84mg

**107-92-6**	Butanoic acid	Sigma Aldrich Company	100μL/95.0mg
**503-74-2**	3-Methylbutanoic acid	Sigma Aldrich Company	100μL/92.5mg
**109-52-4**	Pentanoic acid	Sigma Aldrich Company	100μL/93.0mg
**120-72-9**	Indole	Sigma Aldrich Company	80.5mg (solid)
**83-34-1**	3-Methylindole	Sigma Aldrich Company	45mg (solid) in 75mL methanol

Table S28 Solution 2: compounds used for standards, purity, supplier, and volume/masses used. *Unless stated otherwise.

Calculations for the quantification of compounds:-

To obtain the peak areas for each compound and the ^{13}C equivalent we obtained a separate trace for the single ion of interest and obtained the peak area from this trace. For example the primary m/z product for 3-methylbutanoic acid is 87 and 88 for the ^{13}C isotope. Knowing the mass of ^{13}C compounds added to the sample we can calculate the proportion of ^{13}C compound recovered from the headspace using the calibration graphs constructed. We can then use the ratio of ^{13}C compound added to the sample versus the amount of ^{13}C compound recovered from the headspace to correct the peak area of the compound being quantified and therefore, quantify the amount in the stool samples. To demonstrate the calculations the example of 3-methylbutanoic acid will be used. Peak area units are marked as PAU.

- The calibration curve (figure 1) gave equations y=74.58x and y=66.62x for 3-methylbutanoic acid and ^{13}C 3-methylbutanoic acid respectively.
- Since 0.5μL of solution 1 was used for the calibration curve but 1μL of solution 1 was added to the stool the first step was to calculate the theoretical peak area for 100% recovery of ^{13}C 3-methylbutanoic acid from the sample. In this example 66.62 (slope of ^{13}C 3-methylbutanoic acid) x 0.925 (mass (μg) in 1 μL solution 1) = 61.62 PAU.
- 61.62 PAU is then divided by the PAU of ^{13}C 3-methylbutanoic acid recovered from the sample; which in this case is 0.78 PAU so 61.62PAU / 0.78PAU = 79; this value is the correction factor that allows the calculation of the amount of compound within the stool.
- The correction factor is then multiplied by the peak area of the compound to be quantified from the sample, in this case the peak area of 3-methylbutanoic acid was 0.18PAU, thus 79 x 0.18PAU = 14.21PAU.
- So the peak area for the amount of 3-methylbutanoic acid contained in the stool is 14.21PAU, this can then be used with the calibration curve for 3-methylbutanoic acid (y=74.58x) where y=peak area and x=mass of compound to solve for x so in our example x= 14.21PAU/74.58= 0.19μg
- Finally, 0.19 μg is divided by the mass of stool (3g) = 0.063 μg/g which we then convert to ng/g = 63.3 ng/g.
